# PTBP2 promotes cell survival and autophagy in chronic myeloid leukemia by stabilizing BNIP3

**DOI:** 10.1038/s41419-025-07529-9

**Published:** 2025-03-20

**Authors:** Bibhudev Barik, Shristi Lama, Sajitha IS, Sayantan Chanda, Sonali Mohapatra, Sutapa Biswas, Ghanashyam Biswas, Soumen Chakraborty

**Affiliations:** 1 Cancer Biology Group, Institute of Life Sciences, Bhubaneswar, India; 2 Regional Centre for Biotechnology, Faridabad, India; 3 Department of Veterinary Pathology, Kerala Veterinary & Animal Sciences University, Wayanad, Kerala India; 4 Department of Medical Oncology/Hematology, All India Institute of Medical Sciences, Bhubaneswar, India; 5 Sparsh Hospital and Critical Care, Bhubaneswar, India

**Keywords:** Chronic myeloid leukaemia, Macroautophagy

## Abstract

Polypyrimidine tract binding protein 2 (PTBP2) regulates alternative splicing in neuronal, muscle, and Sertoli cells. PTBP2 and its paralog, PTBP1, which plays a role in B-cell development, was found to be expressed aberrantly in myeloid leukemia. Genetic ablation of Ptbp2 in the cells resulted in decreased cellular proliferation and repopulating ability, decreased reactive oxygen species (ROS), and altered mitochondrial morphology. RNA immunoprecipitation followed by sequencing (RIP-seq) and functional assays confirmed that PTBP2 binds to Bcl-2 Interacting Protein 3 (Bnip3)-3’UTR and stabilizes its expression. Our study also suggests that PTBP2 promotes autophagy, as evidenced by the low levels of LC3-II expression in Ptbp2-knockout cells treated with Bafilomycin A1. This effect was restored upon overexpression of Bnip3 in the knockout cells. Notably, when KCL22-NTC cells were subcutaneously injected into the flanks of mice, they gave rise to malignant tumors, unlike Ptbp2-KO-KCL22 cells. Also, transplantation of KCL22 cells through the tail vein in NOD/SCID mice resulted in higher cell engraftment and increased infiltration of malignant cells in the extramedullary organs. Our study underscores the role of PTBP2 in promoting cell proliferation and tumor formation while enhancing autophagy through Bnip3, thereby supporting the role of PTBP2 as an oncogene in CML.

## Introduction

Chronic myeloid leukemia (CML) is a type of blood cancer that arises due to a genetic translocation between chromosomes 9 and 22, leading to the expression of a constitutively active tyrosine kinase oncogene called BCR::ABL1 [1]. The resultant oncoprotein triggers pro-survival signaling pathways in CML cells, providing them with a proliferative advantage and resistance to apoptosis. While tyrosine kinase inhibitors (TKIs) have significantly improved the survival rate of patients with chronic phase CML, the survival rate is much lower for those in the blast phase [2]. Additionally, most patients require ongoing maintenance therapy for the rest of their lives, as current treatments only suppress the growth of cancer cells. Various BCR::ABL1-dependent and -independent pathways, such as kinase domain mutations, BCR::ABL1 amplification, and aberrant activation of the PI3K and RAS/MAPK signaling pathways, increase the risk of progression and predict poorer response to TKIs [3–5]. Therefore, identifying the factors responsible for disease progression is crucial to enhance the treatment of CML, particularly in advanced cases. Recent studies have highlighted the crucial role of RNA-binding proteins (RBPs) in cancer by regulating mRNA expression. The polypyrimidine tract-binding protein (PTBP) is one such RBP that plays a critical role in regulating mRNA stability, protein expression, and exon exclusion, thereby influencing cellular growth and development [6]. PTBP2, a subtype of PTBP, exhibits differential tissue expression and is correlated with several types of cancer, including glioblastoma, osteosarcoma, and colorectal cancer [7–10]. Autophagy, a cellular process that maintains cellular balance and prevents the accumulation of impaired proteins and organelles, has also been implicated in tumorigenesis [11–13]. Its role in CML is complex, with studies suggesting that it can contribute to both the survival and proliferation of CML cells and the development of drug resistance [14–16].

This study investigated the role of PTBP2 in regulating CML pathology and found that it promotes cell proliferation through oxidative phosphorylation (OXPHOS) supported by mitochondrial fusion. Additionally, PTBP2 promotes autophagy by binding and stabilizing Bnip3, which has been implicated in cancer cell subpopulations and can be a potential therapeutic target. Mice injected with cells that were positive for PTBP2 developed significantly larger tumors and showed increased markers for both proliferation and autophagy. Additionally, a more significant percentage of engraftment and infiltration of malignant cells was observed in mice transplanted with PTBP2-positive cells. This supports the role of PTBP2 as an oncogene in CML.

## Materials and methods

### Cell lines and patient samples

The CML (KCL22, KU812, K562, KYO1, and LAMA84), AML (TF1, HEL, HNT34, and F36P) cell lines, and HEK293T cells were cultured in RPMI medium 1640 (Cat.No. 31800022, Thermo Fisher Scientific Inc., Waltham, MA, USA) +10% fetal bovine serum (Cat.No.10270106, Thermo Fisher Scientific Inc., Waltham, MA, USA) and Dulbecco’s modified Eagle medium (Cat.No.12100046, Thermo Fisher Scientific Inc., Waltham, MA, USA) respectively, with no antibiotic supplements. All cell lines were mycoplasma negative. Blood samples were collected from CML patients, and the study was approved by the institutional human ethical committee (16/HEC/12).

Further detailed information on experimental methods and materials is available in the Supplementary file.

## Results

### Establishment and characterization of PTBP2 knockout CML and AML cells

**Fig. 1:**
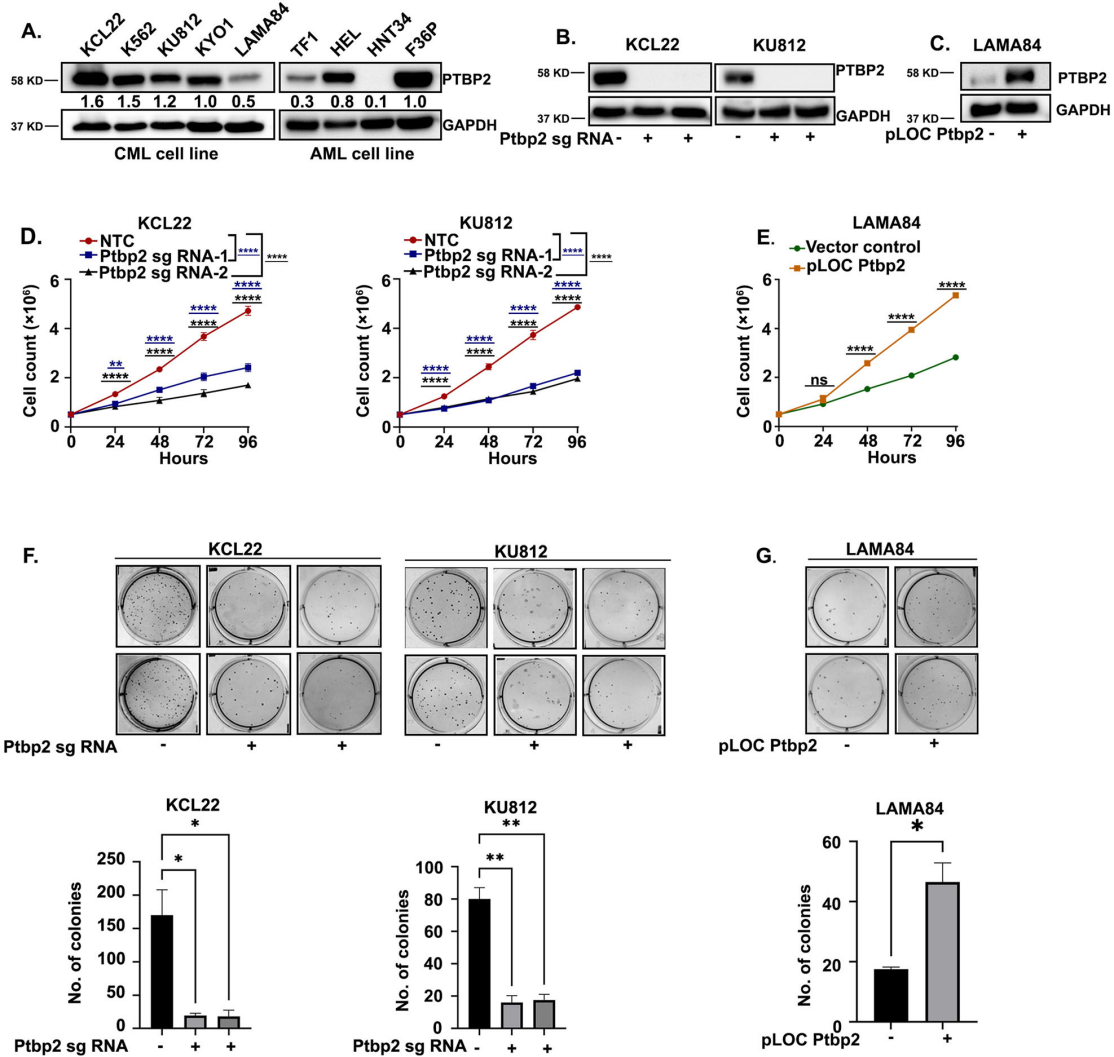
PTBP2 protein expression in different cell types and phenotypic assessment. **A** PTBP2 protein expression in CML cell lines (KCL22, K562, KU812, KYO1, and LAMA84) (lanes 1–5) and AML cell lines (TF1, HEL, HNT34, and F36P) (lanes 6–9). An equal amount of protein (30 µg) was loaded, and GAPDH western blotting was used as an internal loading control. Densitometry quantification is shown in the figure. **B** Western blot analysis of KCL22-NTC and Ptbp2-KO-KCL22, KU812-NTC, and Ptbp2-KO-KU812 cell lysates probed with the indicated antibody. The GAPDH antibody was used as an internal loading control. **C** Western blot of PTBP2 transduced and stably selected LAMA84 (Ptbp2-OE-LAMA84), and vector control cell lysate was probed with the indicated antibody. The GAPDH antibody was used as an internal loading control. **D** The proliferation rates of NTC KCL22-NTC, KU812-NTC, and Ptbp2-KO-KCL22, Ptbp2-KO-KU812 cells were evaluated using the trypan blue exclusion test for cell viability. Proliferation rates at 24, 48, 72, and 96 h are represented in the line graph. Each point represents the mean and standard deviation of independent triplicates (*n* = 3), **p < 0.01 and ****p < 0.0001. **E** LAMA84-vector control and Ptbp2-OE-LAMA84 cell proliferation rate were assessed using a trypan blue dye exclusion test for cell viability. Proliferation rates at 24, 48, 72, and 96 h are represented in the line graph. Each point represents the mean and standard deviation of independent triplicates (*n* = 3), ns: not significant, and ****p < 0.0001. **F** Soft agar colony formation assay was performed using KCL22-NTC, KU812-NTC, Ptbp2-KO-KCL22, and Ptbp2-KO-KU812 cells. Colony numbers are represented in the bar graph below the respective figure. The bar diagram shows the mean value and corresponding standard deviation for the represented data: (*n* = 2) *p < 0.05 and **p < 0.01. **G** Soft agar colony formation assay was performed using LAMA84-vector control and Ptbp2-OE-LAMA84 cells. Colony numbers are represented in the bar graph below the figure. The bar diagram shows the mean value and corresponding standard deviation for the represented data; (*n* = 2) *p < 0.05.

In this study, we examined PTBP2 expression in CML and AML cell lines. The expression of PTBP2 was nearly identical in the four CML cell lines, KCL22, K562, KU812, and KYO1 (Fig. 1A). In one CML cell line, LAMA84, PTBP2 expression was found to be reduced (Fig. 1A). TF1 (human erythroleukemia), HEL (human erythroid leukemia), and F36P (MDS-RAEB) showed varying levels of PTBP2 expression, whereas HNT34 (BCR::ABL1+ human Acute Myeloid Leukemia) cells showed almost no PTBP2 expression among the four AML cell lines (Fig. 1A).

Next, we assessed cell proliferation and colony formation ability in soft agar by ablating Ptbp2 in CML and AML cells. Previously, we reported that the knockdown of Ptbp2 facilitated the reduction in cellular growth and increased apoptosis [17]. Herein, Ptbp2 knockout (KO) was achieved using two distinct gRNAs, as outlined in the Materials and Methods, and several single-cell knockout clones for each cell line were generated and propagated. Using RT-qPCR and western blotting, we confirmed the extent of Ptbp2 knockout in KCL22, KU812, and TF1 cells (Fig. 1B and Supplementary Fig. 1A, B) with respect to the non-targeting control (NTC) cells. Additionally, Ptbp2 overexpression (OE) was confirmed in the LAMA84 cells (Fig. 1C and Supplementary Fig. 1C). To determine the proliferation rate of the cells, we conducted a trypan blue dye exclusion test on both NTC and Ptbp2-KO-KCL22, -KU812, -TF1 cells. As previously noted, the growth rate was significantly decreased in the Ptbp2-KO-KCL22, -KU812, and -TF1 clones (Fig. 1D and Supplementary Fig. 1D). A significant increase in the proliferation rate was observed upon overexpression of Ptbp2 in LAMA84 cells (Fig. 1E). Furthermore, the colony count was also found to be lower in the Ptbp2-KO-KCL22, -KU812, and -TF1 cells than in the NTC cells (Fig. 1F and Supplementary Fig. 1E). PTBP2 overexpressed LAMA84 cells showed a higher number of colonies than vector control cells (Fig. 1G). The bar graphs shown in Fig. 1F, G and Supplementary Fig. 1E indicate a significant change in the colony count.

### PTBP2 targets, stabilizes, and regulates Bnip3 in CML cells

**Fig. 2:**
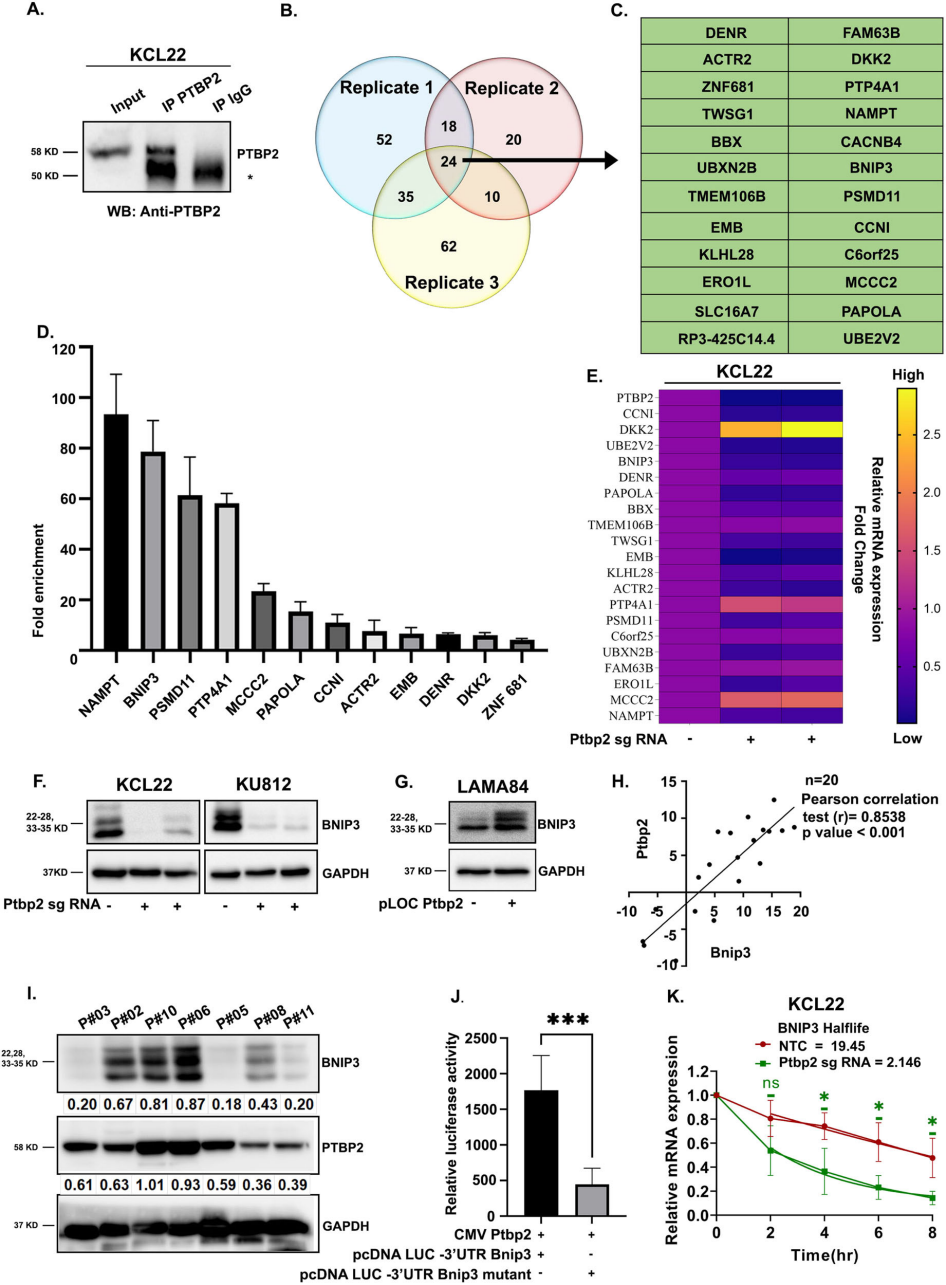
PTBP2 binds and regulates BNIP3. **A** Immunoprecipitation of PTBP2 was performed using a PTBP2 antibody; 10% of the whole-cell lysate was used as input lysate (lane 1), lysate immunoprecipitated with PTBP2 antibody (lane 2), lysate immunoprecipitated with IgG as a negative control (lane 3). The blot was probed with a PTBP2 antibody. “*” represents the IgG. **B** Each circle represents the targets obtained from the single RIP-sequence experiment. Three repeats are presented in the Venn diagram. **C** The common PTBP2-bound targets are represented in the table. **D** RT-qPCR analysis of the PTBP2-bound target transcripts showing a fold enrichment with IgG control. **E** Heat map showing a fold change in relative mRNA expression of the targets in KCL22-NTC and Ptbp2-KO-KCL22 cells. **F** Whole-cell lysates of KCL22-NTC, KU812-NTC, and Ptbp2-KO-KCL22 and Ptbp2-KO-KU812 cells were collected, immunoblotting was performed with BNIP3 antibody, and GAPDH was used as an internal loading control. **G** Whole-cell lysate of LAMA84-vector control and Ptbp2-OE-LAMA84 cells were collected, immunoblotting was performed with BNIP3 antibody, and GAPDH was used as an internal loading control. **H** Ptbp2 and Bnip3 mRNA correlation in CML patient samples (*n* = 20) *p* < 0.001. **I** Immunoblotting of some patient samples probed with BNIP3, PTBP2, and GAPDH antibodies. Densitometric quantification is shown in the figure. **J** 3′UTR binding site in Bnip3 and the mutated site was cloned in the luciferase vector, and luciferase assay was performed by transfecting the clone along with wild-type PTBP2 in HEK293T cells. Renilla luciferase was used as an internal control. After 24 h, luciferase activity was measured. Data presented are the fold change relative to empty vector-transfected cells. The data was analyzed and represented by a bar diagram. ***p < 0.001. **K** Half-life of the BNIP3 expression is represented for KCL22-NTC and Ptbp2-KO-KCL22 cells(*n* = 3) ns: not significant, and *p < 0.05.

PTBP2 functions as an RBP and its four RNA-binding domains regulate the pre-mRNA splicing, translation, and stability of target mRNAs. Therefore, we performed RNA immunoprecipitation and sequencing (RIP-seq) in the KCL22 cell line, which endogenously expresses high levels of PTBP2. First, the complex containing PTBP2 protein and its associated RNA was immunoprecipitated with a PTBP2-specific antibody (Fig. 2A), and subsequently, RNA sequencing was performed to profile the PTBP2-bound transcripts. Peak calling with RIPSeeker was performed on each IP alignment file, which produced a list of peaks and their corresponding candidate transcripts. Bound transcripts showing the maximum read counts were considered. Combining three biological repeats, we found that 24 mRNAs were enriched in the PTBP2 RNA-IP samples compared to the control IgG-IP samples (Fig. 2B, C). Gene Ontology Biological Process analysis of these mRNAs was performed to explore the signaling pathways that PTBP2 might control in CML cells. The Cluster Profiler Bioconductor package was used for gene ontology enrichment of UP- and DOWN-regulated genes for all comparisons with a p-value cutoff of less than or equal to 0.05. The top 10 enriched GO terms were visualized as dot plots (Supplementary Fig. 2A). GO analysis identified significant pathways that regulate transmembrane transport, transmembrane ion transport, and membrane potential, which consists of mitochondrial membrane potential. The mRNA candidates observed in this group were Nampt, Bnip3, and Cacnb4. The binding efficiency between the mRNAs and PTBP2 was validated by independent RNA-IP experiments followed by RT-qPCR, confirming the association between PTBP2 and its targets. Of the 24 targets, strong binding efficiency was detected in 11 (Fig. 2D). The remaining 13 exhibited no notable changes. Nampt, followed by Bnip3, showed the highest binding efficiency. The expression levels of 24 targets were compared by RT-qPCR between KCL22-NTC and Ptbp2-KO-KCL22 cells and are represented in a heat map (Fig. 2E). Although the binding affinity to Nampt was the highest, no corresponding change in NAMPT protein expression was detected between KCL22-NTC and Ptbp2-KO-KCL22 cells (figure not shown). Thus, the 2nd top target, Bnip3 (Bcl-2 Interacting Protein 3), was considered, and its role was further explored in the pathology of CML.

The role of the BH3-only protein BNIP3 in cancer is controversial. Increased BNIP3 levels in cancer patients have been linked to good as well as poor prognosis, as BNIP3 contributes to both pro-cell death and pro-survival signals [18, 19]. RT-qPCR and western blot results illustrated that the expression of BNIP3 in KCL22-, KU812-, and TF1- NTC cells was higher than that in Ptbp2-KO cells (Fig. 2F and Supplementary Fig. 2B). An increase in the expression of Bnip3 was noted when PTBP2 was overexpressed in LAMA84 cells (Fig. 2G and Supplementary Fig. 2C). Analysis of a publicly available dataset GSE4170, showed that as the disease progressed, there was a gradual increase in the expression of both the genes (Supplementary Fig. 2D) [20]. An independent set of CML samples (*n* = 20) also showed a positive Pearson correlation (*r*) of 0.8538 (*p* < 0.001) between Ptbp2 and Bnip3 expression (Fig. 2H). Western blot analysis of some patient samples showed the same correlation (Fig. 2I). Thus, our study revealed that Ptbp2 strongly correlates with Bnip3 in CML.

As PTBP2 is an RBP, and Bnip3 is one of the most enriched targets in RIP sequencing as well as RT-qPCR validation, we wanted to look into the binding site(s) of PTBP2 on Bnip3 mRNA, for which we scanned Bnip3 mRNA using beRBP (https://bioinfo.vanderbilt.edu/beRBP/predict.html). Only one specific PTBP2 binding site (CUUUUCU) was observed in the 3′-UTR, which had a binding score of 0.362 (Supplementary Fig. 2E). As we observed downregulation of Bnip3 in the absence of PTBP2, we speculate that PTBP2 binds to the 3’UTR of Bnip3 to stabilize it. Next, to confirm the binding of PTBP2 to Bnip3, we cloned the predicted site into a luciferase vector. Transfection with recombinant Ptbp2 (kindly gifted by Dr. Miriam Llorian, University of Cambridge, Cambridge, UK) increased the stability of the relative luciferase reporter count compared to the empty vector control. Modification of the binding site (CAAAACA) using site-directed mutagenesis decreased the relative luciferase reporter count, providing evidence that PTBP2 binds to and regulates the stability of Bnip3 (Fig. 2J and Supplementary Fig. 2E). The analysis of BNIP3 mRNA half-life as a measure of mRNA stability was investigated after transcriptional inhibition with Actinomycin D. We observed a noticeable impact on the stability of BNIP3 when PTBP2 was knocked out from the cells (Fig. 2K). Thus, our findings indicate that PTBP2 binds and functions through BNIP3.

### PTBP2-mediated oxidative phosphorylation supported by mitochondrial fusion increases cell proliferation

**Fig. 3:**
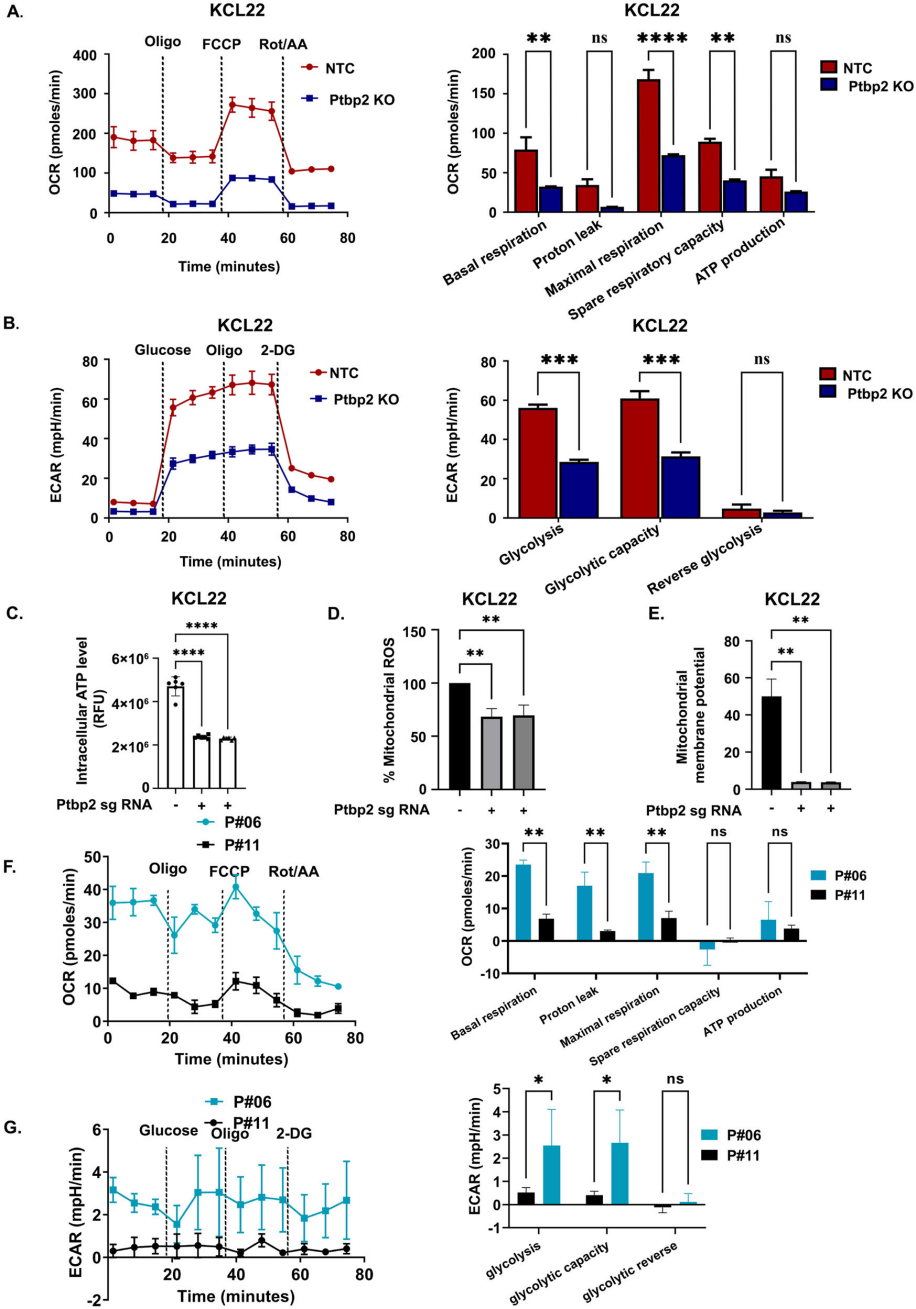
PTBP2 deficiency results in mitochondrial dysfunction. **A** The Seahorse XFp Cell Mito Stress test was utilized, and the OCR measurement was examined in KCL22-NTC vs. Ptbp2-KO-KCL22 cells treated sequentially with oligomycin, FCCP, and rotenone (left panel). The outcome represents the mean and standard deviation of the experiment. Two-way ANOVA was used for statistical analysis, and the comparison test (right panel) **p < 0.01, ****p < 0.0001, and ns not significant. **B** The glycolytic ability was measured in KCL22-NTC vs. Ptbp2-KO-KCL22 cells (left panel). The experiments were carried out using the Seahorse XFp Glycolysis Stress test, and the flow chart and bar graph depict the ECAR measurement (right panel). The results represent the mean and standard deviation of the experiment. Two-way ANOVA was implemented for statistical analysis, followed by a multiple comparison test ***p < 0.001 and ns not significant. **C** Intracellular ATP was assessed in KCL22-NTC vs. Ptbp2-KO-KCL22 cells. The ATP luminescence signals were normalized to the quantity of protein (*n* = 4), ****p < 0.0001. **D** Flow cytometry assessed Mitochondrial superoxide distribution using MitoSOX dye and displayed by a bar graph in KCL22-NTC vs. Ptbp2-KO-KCL22 cells, (*n* = 2), **p < 0.01. **E** Flow cytometry measurement of MMP using JC-1 dye in KCL22-NTC vs. Ptbp2-KO-KCL22 cells, illustrated by a bar graph, **p < 0.01, compared to the controls. **F** The Seahorse XFp Cell Mito Stress test was utilized, and the OCR measurement was examined in the primary cell (P#06) vs. (P#11) treated sequentially with oligomycin, FCCP, and rotenone (left panel). The outcome represents the mean and standard deviation of the experiment. Two-way ANOVA was used for statistical analysis, and the comparison test (right panel), **p < 0.01, and ns not significant. **G** The glycolytic ability was measured in the primary cell (P#06) vs. (P#11) (left panel). The experiments were carried out using the Seahorse XFp Glycolysis Stress test, and the flow chart and bar graph depict the ECAR measurement (right panel). The results represent the mean and standard deviation of the experiment. Two-way ANOVA was implemented for statistical analysis, followed by a multiple comparison test *p < 0.05 and ns not significant.

As cell proliferation is directly linked to cellular energy metabolism, we investigated the mitochondrial function (i.e., OCR) and substrate-level phosphorylation via glycolysis (i.e., ECAR) of KCL22-NTC and Ptbp2-KO-KCL22 cells and LAMA84-vector control and Ptbp2-OE-LAMA84 cells using a Seahorse XFp extracellular flux analyzer. The basal respiration rate and ATP production were higher in the KCL22-NTC cells compared to the Ptbp2-KO cells respectively (Fig. 3A), and lower in the LAMA84-vector control cells compared to the Ptbp2-OE-LAMA84 cells (Supplementary Fig. 3A). The spare respiratory capacity, defined as the difference between maximal and basal respiration, was found to be decreased by 55% in Ptbp2-KO-KCL22 cells. Furthermore, a similar decrease in the glycolysis rate was observed in the Ptbp2-KO-KCL22 compared to the KCL22-NTC cells (Fig. 3B) and decreased in the LAMA84-vector control compared to Ptbp2-OE-LAMA84 cells (Supplementary Fig. 3B). Ptbp2-KO-KCL22 and KU812 cells showed reduced intracellular ATP levels (Fig. 3C and Supplementary Fig. 3C). As expected, intracellular ATP levels increased in Ptbp2-OE-LAMA84 cells (Supplementary Fig. 3D). Thus, our data suggest that PTBP2 activates both OXPHOS and glycolysis. Respiration is essential for cellular proliferation and is correlated with biological aggressiveness. ROS-regulated signaling pathways are notably upregulated in different types of cancer, leading to cell proliferation, survival, and other factors contributing to cancer onset and progression [21]. In addition to decreased ATP production and OXPHOS activity, lower levels of mitochondrial ROS were observed in Ptbp2-KO-KCL22, Ptbp2-KO-KU812 cells, and LAMA84-vector control cells (Fig. 3D, Supplementary Fig. 3E, F), including the lower level of mitochondrial membrane potential in the Ptbp2-KO-KCL22 cells (Fig. 3E). We also examined OCR and ECAR in primary cells. Two primary cells (patient sample) were analyzed: P#06 (patient sample 06), which exhibited elevated levels of PTBP2 and BNIP3, and P#11 (patient sample 11), with low levels of both PTBP2 and BNIP3 in comparison to P#06 (data presented in Fig. 2I). Our primary sample data aligned with the findings from our in-vitro cell line studies (Fig. 3F, G). Live cell imaging showed a change in the membrane potential (Supplementary Fig. 3G).

**Fig. 4:**
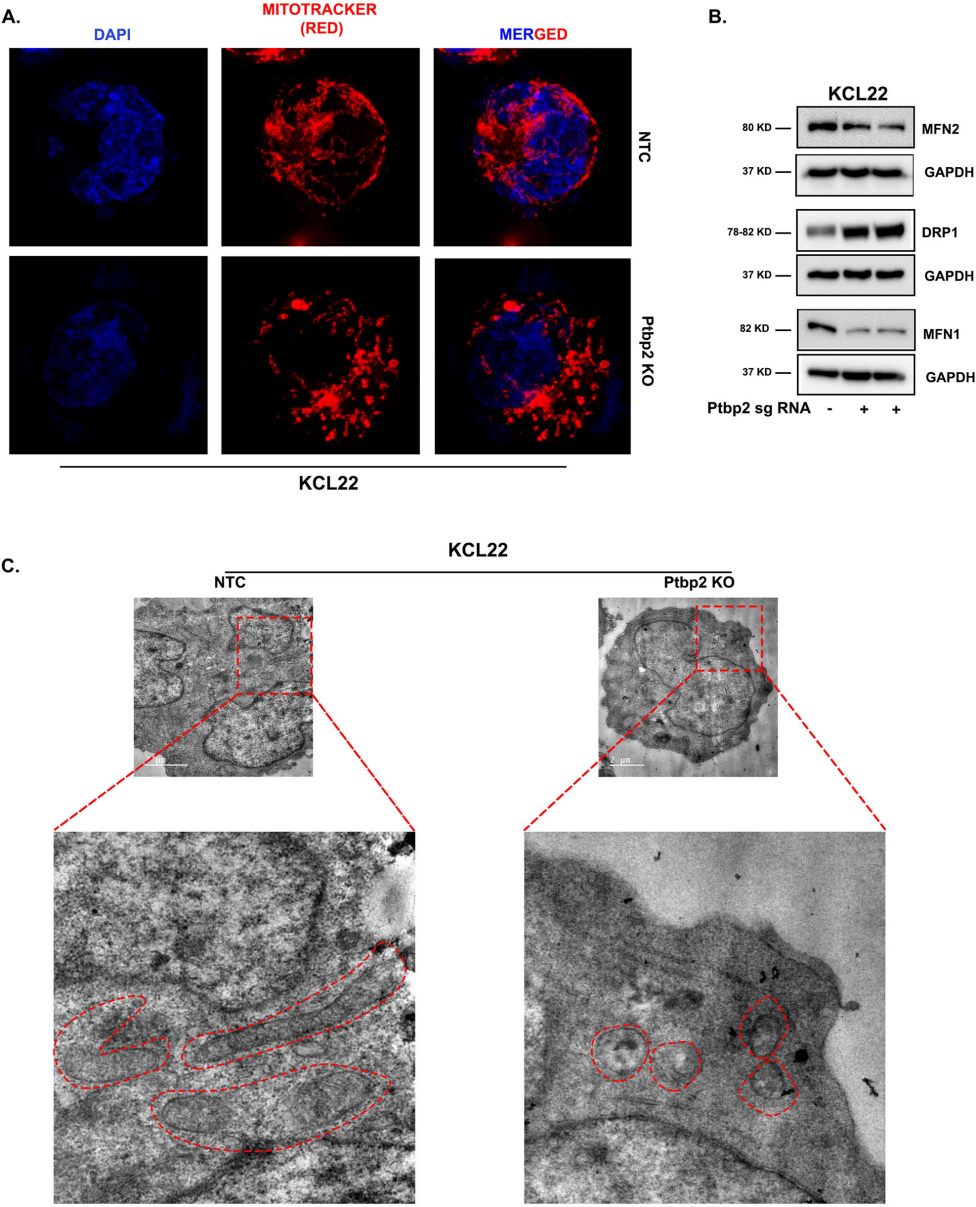
Diminution of PTBP2 affects mitochondrial morphology. **A** Confocal microscopy image of KCL22-NTC vs. Ptbp22-KO-KCL22 cells using CMXros mitotracker red dye. **B** Western blot analysis of MFN2, DRP1, and MFN1 in control KCL22-NTC vs Ptbp2-KO-KCL22 cells. An equal amount of protein (30 µg) was used for both cell lines, and GAPDH was used as an internal loading control. **C** Representative TEM images of mitochondria in KCL22-NTC and Ptbp2-KO-KCL22 cells.

Mitochondrial fusion and fission play essential roles in mitochondrial morphology, and recent studies suggest that increased fusion correlates with increased Mitofusin- 1 and 2 (MFN1 and MFN2) that boosts OXPHOS, leading to heightened cell proliferation [22]. Thin and elongated mitochondria were observed under a confocal microscope in KCL22-NTC cells using MitoTracker staining, whereas, in Ptbp2-KO cells, we observed dotted or fragmented mitochondria (Fig. 4A, upper panel, and lower panel, respectively). For further confirmation, we checked the expression of MFN1 and MFN2, which was seen to be reduced in the KO condition (Fig. 4B). We also examined the expression of dynamin-related protein 1 (DRP1), which drives constriction and fission during mitochondrial division. The Ptbp2-KO-KCL22 cells showed higher expression of DRP1 with respect to the KCL22-NTC cells (Fig. 4B). Thin and elongated mitochondria were also observed in the Ptbp2-OE-LAMA84 cells. In contrast, the vector control cells showed only dotted and fragmented mitochondria (Supplementary Fig. 4A). Likewise, an increase in MFN1 and MFN2 expression and a decrease in DRP1 expression was observed in Ptbp2-OE-LAMA84 cells in comparison to vector control cells (Supplementary Fig. 4B). The transmission electron microscope has been very useful in studying the mitochondrial structure. Intact rod-shaped elongated mitochondria were observed in KCL22-NTC cells; however, damaged mitochondria were observed in Ptbp2-KO-KCL22 cells (Fig. 4C). Thus, PTBP2 promotes increased OXPHOS and mitochondrial fusion and thereby facilitates cell proliferation.

### The presence of PTBP2 promotes autophagy through BNIP3

**Fig. 5:**
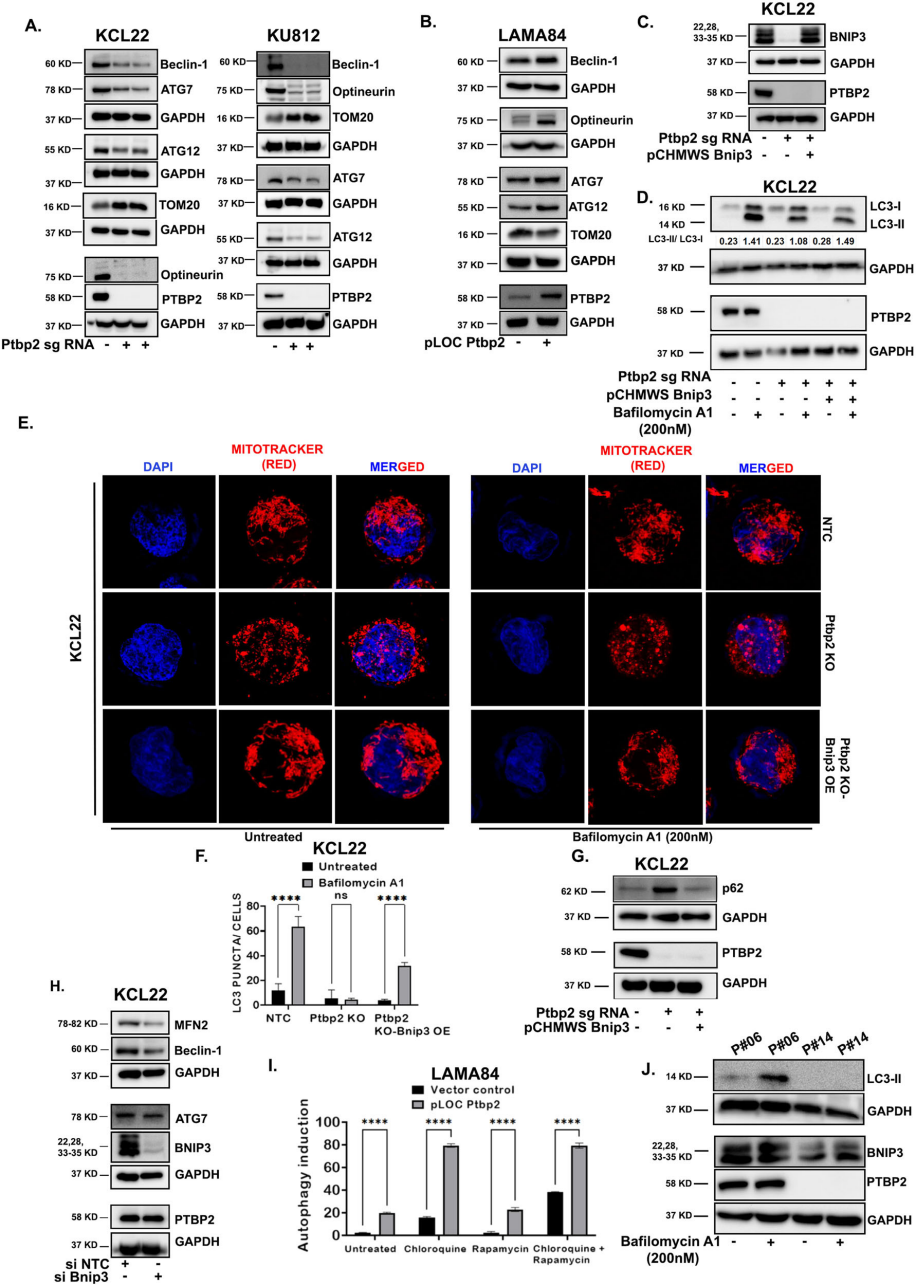
Regulation of autophagy by PTBP2 in CML cells. **A** Western blot analysis of Beclin-1, ATG7, ATG12, Optineurin, TOM20, and PTBP2 in KCL22-NTC vs. Ptbp2-KO-KCL22 cells (left panel) and in KU812-NTC vs Ptbp2-KO-KU812 cells (right panel). An equal amount of protein (30 µg) was loaded and GAPDH was used as an internal loading control. **B** Representative immunoblotting analysis of Beclin-1, ATG7, ATG12, Optineurin, TOM20, and PTBP2 in vector control LAMA84 vs Ptbp2-OE-LAMA84 cells. **C** Western blot analysis was performed for BNIP3 in KCL22-NTC, Ptbp2-KO-KCL22, and Ptbp2-KO-Bnip3-OE-KCL22 cells by taking an equal amount of protein. GAPDH was considered a loading control. **D** KCL22-NTC, Ptbp2-KO-KCL22, Ptbp2-KO-Bnip3-OE-KCL22 cells were treated with Bafilomycin A1 (200 nM) for 18 h, lysed and immunoblotted for LC3B with an equal amount of protein (30 µg). A western blot was also performed using the Ptbp2 antibody. GAPDH was used as a loading control. Densitometry quantification is shown in the figure. **E** Confocal microscopic image of KCL22-NTC, Ptbp2-KO-KCL22, and Ptbp2-KO-Bnip3-OE- KCL22 cells using CMXros mitotracker red dye before and after treatment with Bafilomycin A1 (200 nM) for 18 h. **F** Representative bar diagram of LC3 puncta in KCL22-NTC, Ptbp2-KO, and Ptbp2-KO-Bnip3-OE-KCL22 cells treated with Bafilomycin A1 (200 nM) for 18 h. ns not significant and ****p < 0.0001. **G** Western blot analysis was performed for p62 and PTBP2 in KCL22-NTC, Ptbp2-KO-KCL22, and Ptbp2-KO-Bnip3-OE-KCL22 cells by taking an equal amount of protein. GAPDH was considered a loading control. **H** KCL22 cells were transfected with scrambled siRNA and Bnip3 siRNA, and after 48 h, cells were lysed, and immunoblotting was performed for DRP1, MFN2, Beclin-1, ATG7, PTBP2, and BNIP3 by taking an equal amount of protein. GAPDH was used as a loading control. **I** Flow cytometry-based profiling of autophagy in LAMA84-vector control vs. Ptbp2-OE-LAMA84 cells was done. Cells were treated or untreated with 0.5 µM rapamycin, 10 µM chloroquine (CQ), or both for 20 h. After staining with CYTO-ID green detection reagent for 30 mins in the dark, cells were washed and analyzed by flow cytometry. Results were analyzed and represented in a bar diagram. ****p < 0.0001. **J** Immunoblotting for LC3B was performed in two patient samples treated with Bafilomycin A1 (200 nM) for 18 h.

Previous studies have suggested that BNIP3 can bind to Bcl-2 and release Beclin-1. This process promotes autophagy and inhibits apoptosis [23]. In Ptbp2-KO-KCL22 and KU812 cells, the expression of Beclin-1 was decreased, whereas it was increased in LAMA84 cells where Ptbp2 was overexpressed (Fig. 5A, B). The transformation of Microtubule-associated Protein 1 Light Chain 3B (LC3B) from its unconjugated state (LC3-I) to a phosphatidylethanolamine-conjugated state (LC3-II) plays a crucial role in the formation of autophagosomes [24]. BNIP3 is primarily located on the mitochondria and has an LC3B interacting region (LIR) that facilitates the removal of mitochondria via autophagy [25]. ATG7 plays a role in converting LC3-I to LC3-II, and ATG12 plays a crucial role during autophagosome function. Both ATG7 and ATG12 were decreased in Ptbp2-KO-KCL22 and KU812 cells (Fig. 5A). Ptbp2-OE-LAMA84 cells showed upregulation of ATG7 and ATG12 (Fig. 5B). As BNIP3 is also involved in mitophagy, we examined the expression of mitophagy-related genes Optineurin and TOM20. Optineurin, an autophagy receptor in the parkin-mediated mitophagy pathway, was upregulated in KCL22-NTC, KU812-NTC, and Ptbp2-OE-LAMA84 cells compared to their counterparts (Fig. 5A, B), whereas TOM20, whose abnormal expression is considered an indicator of mitophagy, was downregulated in KCL22-NTC, KU812-NTC, and Ptbp2-OE-LAMA84 cells (Fig. 5A, B). PTBP2 and individual GAPDH expression levels are also shown (Fig. 5A, B). Furthermore, we overexpressed Bnip3 in Ptbp2-KO-KCL22 cells to determine the function of BNIP3 (Fig. 5C). To gauge the activity of autophagic flux, we used bafilomycin A1, an inhibitor of autophagosome-lysosome fusion, and studied the conversion of LC3-I to LC3-II. After treatment with Bafilomycin A1, KCL22-NTC cells showed higher expression of LC3-II than LC3-I, almost the same as in Ptbp2-KO-Bnip3-OE-KCL22 cells. The LC3-II/LC3-I ratio was quantified and shown in Fig. 5D (1st panel). Again, on treating cells with Bafilomycin A1, a partial increase in fragmented mitochondria in KCL22-NTC cells along with elongated ones was observed, but no difference in mitochondrial dynamics was observed in Ptbp2-KO-KCL22 cells (Fig. 5E, upper and middle panel, respectively). However, with the same condition, upon BNIP3 overexpression, in the Ptbp2-KO-KCL22 cells, the cells behaved as KCL22-NTC cells in terms of mitochondrial dynamics with a partial increase in elongated mitochondria, suggesting a possible role of BNIP3 in the process (Fig. 5E, lower panel). On Bafilomycin A1 treatment, the number of small LC3 puncta was quantified. There was a significant reduction in the number of puncta in the Ptbp2-KO-KCL22 cells with respect to the KCL22-NTC cells; however, upon overexpression of Bnip3 in the Ptbp2-KO-KCL22 cells, the number of puncta was found to be increased (Fig. 5F and Supplementary Fig. 5A). P62 is an autophagy substrate used as an autophagy activity receptor. The lack of autophagy was evident by the accumulation of p62 only in the Ptbp2-KO-KCL22 cells (Fig. 5G, upper panel) and not in KCL22-NTC and Ptbp2-KO-BNIP3-OE-KCL22 cells. A direct role of PTBP2 was observed when Ptbp2-OE-LAMA84 cells were used with respect to the vector control. The ratio of LC3-II/LC3-I was higher in Ptbp2-OE-LAMA84 cells than in the vector control cells (Supplementary Fig. 5B, lane 4 with respect to lane 2). These results suggested that PTBP2 plays a role in the autophagy of CML cells. Ablation of Mfn2 led to impaired autophagic degradation and suppressed cell proliferation [26, 27]. To understand whether this is mediated through BNIP3, we used a scrambled siRNA and BNIP3-specific siRNA to knock down the expression of BNIP3 in KCL22 cells. Upon successful knockdown of BNIP3, MFN2 was found to be reduced, demonstrating that BNIP3 works through MFN2 (Fig. 5H, 1st panel). In the same lysate, we checked for the expression of Beclin-1 and ATG7, which was found to be reduced. Thus, our data suggest that BNIP3 promotes autophagy through the Beclin-1 pathway (Fig. 5H, 2nd and 3rd panel).

The Cyto-ID autophagy detection kit measures autophagic vacuoles and monitors autophagic flux in lysosomally inhibited live cells. Chloroquine (CQ), an antimalarial drug, inhibits autophagy by preventing the fusion of autophagosomes with lysosomes and slowing lysosomal acidification. Ptbp2-OE-LAMA84 cells, when treated with CQ or combined with rapamycin, showed higher autophagic flux than the vector control cells (Fig. 5I, bars 4 and 8, with respect to bars 3 and 7). The autophagic flux was reduced when only rapamycin was used without chloroquine (Fig. 5I, bars 5 and 6).

We looked for the LC3B turnover in CML patient samples for clinical relevance. The patient sample expressing PTBP2 showed LC3-II expression after treatment with Bafilomycin A1, whereas in the sample that did not express PTBP2, even after Bafilomycin A1 treatment, LC3-II was found to be absent (Fig. 5J). Our observation suggests that PTBP2-mediated stabilization of Bnip3 promotes autophagy and enhances cell survival.

### KCL22 cells promote tumorigenesis in CML mice model

**Fig. 6:**
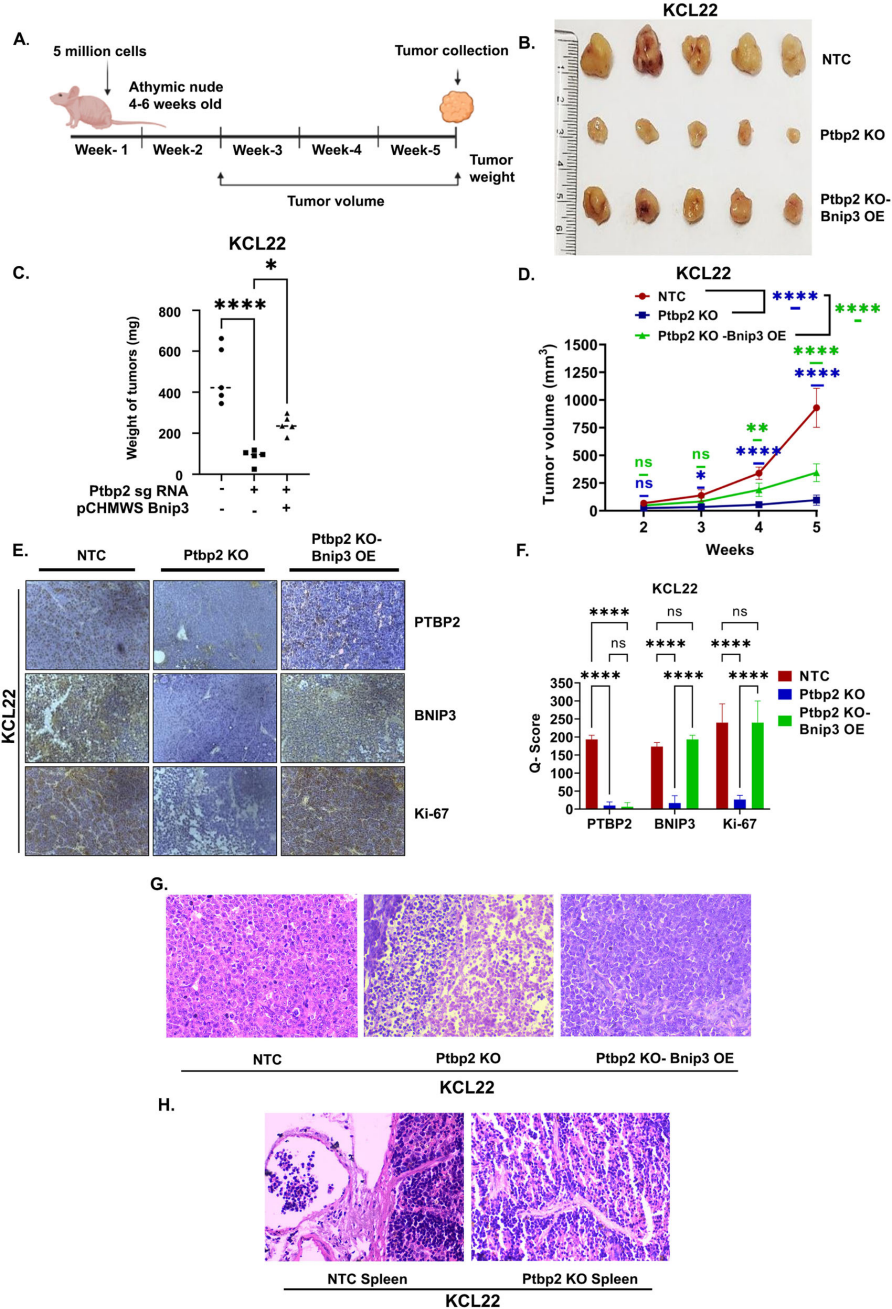
PTBP2 promotes tumor growth. **A** Plan of work for the in vivo study. Five million respective cells were injected subcutaneously in 4- to 6-week-old female athymic nude mice. After 5 weeks, the tumor was collected and processed for IHC. In between that, tumor volume was measured twice per week. The cartoon was created using Biorender. **B** Representative tumor images are represented in the mice injected with KCL22-NTC, Ptbp2-KO-KCL22, and Ptbp2-KO-Bnip3-OE-KCL22 cells. The experiment was performed two times, with 5 animals in each group, and the Vernier caliper was used to measure the volume. **C** Graphical representation of the tumor weight of each group of mice and the average weight of tumors were represented by scatter plot. One-way ANOVA was performed to calculate the statistical significance between those groups. *p < 0.05, and ****p < 0.0001. **D** The rate of tumor progression of KCL22-NTC, Ptbp2-KO-KCL22, and Ptbp2-KO-Bnip3- OE-KCL22 cells is represented by the tumor volume calculated each week. Data are represented by a line graph with an error bar. ns not significant, *p < 0.05, **p < 0.01 and ****p < 0.0001. **E** Representative IHC images showing the PTBP2, BNIP3, and Ki-67 expression pattern in KCL22-NTC, Ptbp2-KO-KCL22, and Ptbp2-KO-Bnip3-OE-KCL22 group tumor. **F** IHC scoring is represented in the bar diagram by calculating the Q score for PTBP2, BNIP3, and Ki-67 (*Q* score = staining intensity (*I*) × percent of staining (*P*)) of KCL22-NTC, Ptbp2-KO-KCL22, and Ptbp2-KO-Bnip3-OE-KCL22 group tumor (*n* = 3). ns not significant and ****p < 0.0001. **G** The tumor tissue was stained with H&E and visualized at ×400. **H** The spleen tissue was stained with H&E and visualized at ×400.

To investigate the effect of PTBP2 in vivo, exponentially growing KCL22-NTC, Ptbp2-KO-KCL22, and Ptbp2-KO-Bnip3-OE-KCL22 cells were subcutaneously injected into the flanks of athymic nude mice. From the second week, tumor volume was measured using a digital caliper. The animal study plan is shown in Fig. 6A. We observed large tumors in mice injected with KCL22-NTC cells compared to those injected with Ptbp2-KO-KCL22 cells (Fig. 6B, upper and middle panel, respectively). However, the introduction of Ptbp2-KO-Bnip3-OE-KCL22 cells resulted in tumors of size between the KCL22-NTC and Ptbp2-KO cells (Fig. 6B, lower panel). Tumor weight and volume were higher in KCL22-NTC and Ptbp2-KO-Bnip3-OE-KCL22 cells than in Ptbp2-KO-KCL22 cells (Fig. 6C and 6D, respectively). In addition, a significant decrease in PTBP2, BNIP3, and Ki-67 expression was observed in the Ptbp2-KO-KCL22 cells with respect to the NTC, as confirmed by IHC (Fig. 6E, 1st and 2nd column). However, upon overexpression of BNIP3 in the Ptbp2-KO-KCL22 cells, expression of Ki-67 and Beclin-1 was found to be increased (Fig. 6E, 3rd column). The Q score was determined by multiplying the proportion of positively stained cells (P) by the staining intensity (I). We observed a significant decrease in the Q score of PTBP2, BNIP3, and Ki-67 in Ptbp2-KO-KCL22 compared to the KCL22-NTC tumor; however, overexpression of BNIP3 in the Ptbp2-KO-KCL22 cells showed a higher Q score (Fig. 6F). In tissue samples derived from the tumors of NTC mice, apparently healthy and actively dividing cells were observed upon H&E staining. The cells were arranged in diffuse sheets supported by well-vascularized fibrous tissue septa. Cellular pleomorphism, a characteristic feature of malignancy, was more evident in mice with KCL22-NTC tumors than in tissue samples derived from tumors of Ptbp2-KO-KCL22 (Fig. 6G). KCL22-NTC cells showed large blasts with scant to moderate cytoplasm. The nuclei were vesicular, round, oval, or sometimes folded, with finely stippled chromatin, prominent nucleoli, and high mitotic activity. Some cells were very large, with a high nuclear-cytoplasmic ratio and prominent nucleoli. In the Ptbp2-KO-KCL22 group, large areas of necrosis were observed with small islands of proliferating neoplastic cells in between. Individual cell necrosis was evident, characterized by highly eosinophilic cytoplasm and loss of nuclei or karyolysis. The neoplastic cells in the active areas were mainly a uniform population of immature cells with some intermediate forms and without much pleomorphism. In the Ptbp2-KO-Bnip3-OE-KCL22 group, the cells were primarily consistent with a lower mitotic rate than in the NTC group but higher than that in the KO group. The migration of KCL22-NTC cells through the subcapsular sinus and trabeculae into the spleen was observed. However, no migration of cells into the spleen was observed in mice injected with Ptbp2-KO-KCL22 (Fig. 6H). Thus, we conclude that high levels of PTBP2 may contribute to developing a more aggressive form of the disease.

**Fig. 7:**
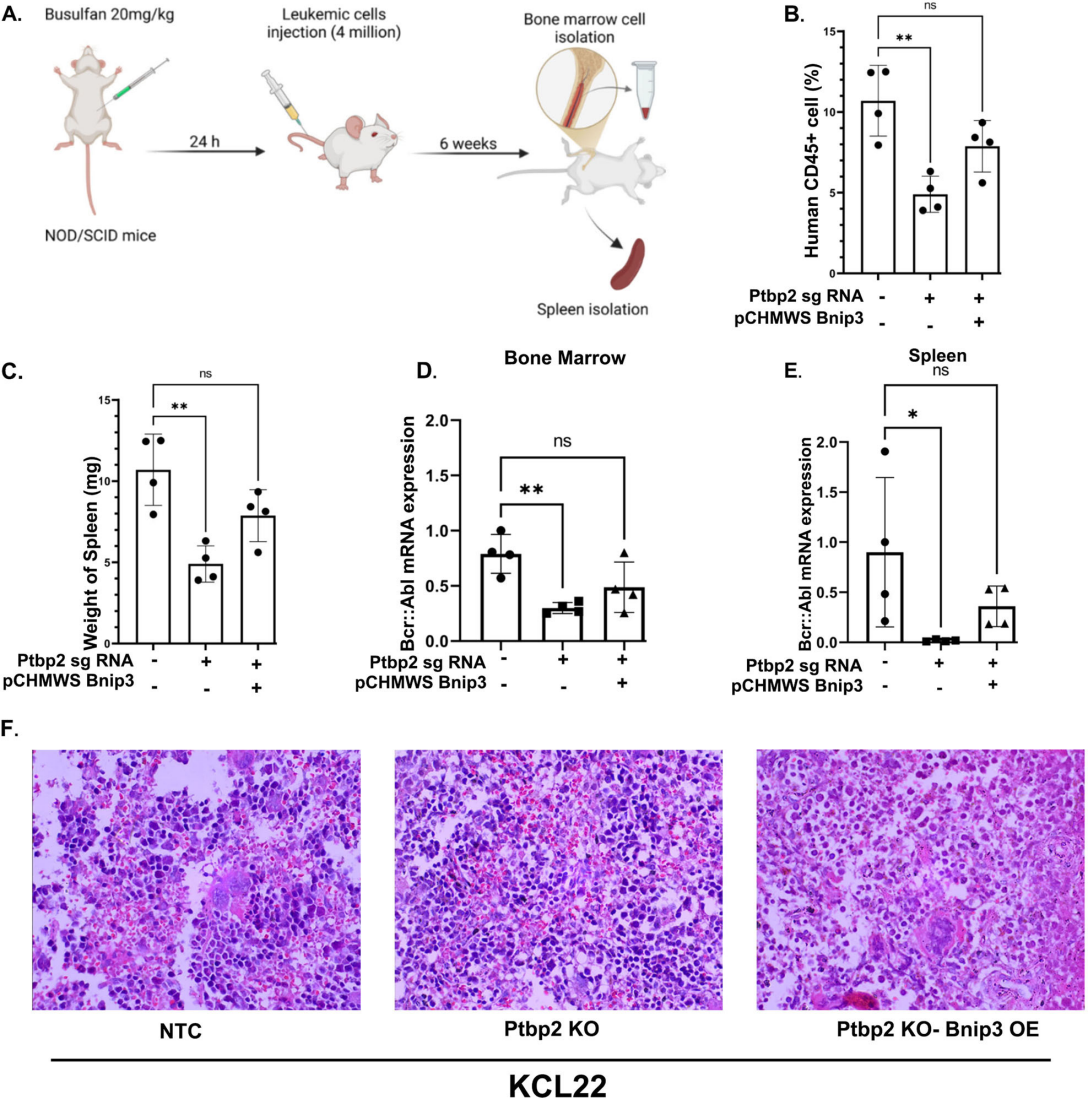
PTBP2 promotes bone marrow engraftment. **A** Work Plan for the NOD/SCID engraftment study. Busulfan (20 mg/kg) was injected intraperitoneally in 4- to 6-week-old female athymic NOD/SCID mice. After 24 h, four million leukemic cells were injected intravenously, with four animals in each group. After 6 weeks, the bone marrow cells and spleen were isolated. The cartoon was created using Biorender. **B** Graphical representation of the average percentage of human CD45+ of each group of mice. One-way ANOVA was performed to calculate the statistical significance between those groups. ns not significant and **p < 0.01. **C** Graphical representation of the spleen weight of each group of mice and the average weight of spleen were represented by scatter plot with bar. One-way ANOVA was performed to calculate the statistical significance between those groups. ns not significant and **p < 0.01. **D** mRNA expression of BCR::ABL1 using RT-qPCR in the bone marrow cells of KCL22-NTC, Ptbp2-KO-KCL22, and Ptbp2-KO-Bnip3-OE-KCL22. One-way ANOVA was performed to calculate the statistical significance between those groups, ns not significant and **p < 0.01. **E** mRNA expression of BCR::ABL1 using RT-qPCR in the spleen cells of KCL22-NTC, Ptbp2-KO-KCL22, and Ptbp2-KO-Bnip3-OE-KCL22. One-way ANOVA was performed to calculate the statistical significance between those groups ns not significant and *p < 0.05. **F** The spleen tissue was stained with H&E and visualized at ×400.

4 × 10^6^ cells from KCL22-NTC, Ptbp2-KO-KCL22, and Ptbp2-KO-Bnip3-OE-KCL22 were transplanted into 4- to 6-week-old male NOD/SCID mice via the tail vein. After 6 weeks, the mice were sacrificed, and their bone marrow was collected while the spleen was preserved in formalin (Fig. 7A). CD45 is a pan-leukocyte marker expressed in hematopoietic cells that enables monitoring of human cell engraftment. A significant decrease in human CD45 expression in the bone marrow was observed in the Ptbp2-KO-KCL22 mice compared to the KCL22-NTC mice, indicating that PTBP2 may play a role in the enhanced engraftment of CML cells. The Ptbp2-KO-Bnip3-OE-KCL22 group showed intermediate levels of hCD45 expression (Fig. 7B). Furthermore, the spleen weight was found to be greater in the KCL22-NTC group compared to the other two groups (Fig. 7C). Additionally, the expression of BCR:: ABL1 was higher in both the bone marrow and spleen of the KCL22-NTC and Ptbp2-KO-Bnip3-OE-KCL22 compared to the Ptbp2-KO-KCL22 (Fig. 7D, E). The spleen of KCL22-NTC, Ptbp2-KO-KCL22, and Ptbp2-KO-Bnip3-OE-KCL22 group mice were analyzed for the presence of extramedullary hematopoiesis. In the KCL22-NTC group, the spleen showed increased extramedullary hematopoiesis characterized by the expansion of the red pulp with hematopoietic cells of multiple lineages but with a predominance of myeloid progenitor cells. Large megakaryocytes were also observed (Fig. 7F, left). The presence of large numbers of immature erythroid cells, including nucleated RBCs and myeloid cells, showed a left shift, and thus, the presence of more immature forms was observed in the red pulp. The extramedullary hematopoiesis observed was differentiated from splenitis/inflammatory foci by the relative absence of vascular changes and very few mature neutrophils/lymphocytes in the red pulp. A similar histological picture was seen in the Ptbp2-KO-Bnip3-OE-KCL22 group, with fewer immature cells than the KCL22-NTC group (Fig. 7F, right). In the Ptbp2-KO-KCL22 group, the organs showed no overt extramedullary hematopoiesis and predominantly mature hematopoietic cells, and a very small number of megakaryocytes were observed (Fig. 7F, middle). Thus, PTBP2 appears to function as an oncogene, contributing to the progression of CML and facilitating the infiltration of cells into extramedullary organs.

## Discussion

RBPs, that include Musashi2, heterogeneous nuclear ribonucleoprotein H1, hnRNPA1, and others, have been implicated in CML [28–32]. Msi2 expression was significantly increased, followed by Ptbp2 expression, in blast crisis samples compared to other RBPs [17]. PTBP2 has been associated with oncogenic RNA splicing in various cancers, such as glioblastoma, osteosarcoma, and colorectal cancer [7, 8, 33–35]. Knockdown of both PTBP1 and PTBP2 slowed glioma cell proliferation [36], and PTBP2 null mice died shortly after birth and exhibited misregulation of alternative splicing in genes involved in cytoskeletal remodeling and cell proliferation [37, 38]. We found that the knockout of Ptbp2 in several CML and AML cells reduced proliferation and long-term colony formation ability. We demonstrated that PTBP2 could increase glycolysis and OXPHOS, leading to a more significant proliferation of cells through increased ATP production and possibly elevated macromolecular precursors. Yao et al. [22] recently reported that glycolysis and mitochondrial respiration support the proliferation of oncogene-transformed cells, which aligns with our observation. MFN1 and MFN2 are proteins found in the outer membrane of mitochondria that primarily function as a mitochondrial fusion protein. Mitochondrial biogenesis, as assessed by fusion, was increased in CML cells, which was counteracted when Ptbp2 was knocked out. The elongated shape of the mitochondria in KCL22-NTC cells and the presence of MFN1/MFN2 suggest that excessive fusion of mitochondria drives cell proliferation. DRP1 is the primary regulator of mitochondrial fission. In Ptbp2-KO-KCL22 cells, the existence of DRP1 alongside dotted mitochondria suggests excess fission. Increased mitochondrial fission in Ptbp2 conditional KO (cKO) spermatids was shown due to increased DRP1. This leads to differences in the number and shape of mitochondria between WT and Ptbp2 cKO spermatids [39].

Mitochondria, the primary source of ROS in most mammalian cells, are typically higher in cancer cells and function as crucial signaling molecules regulating autophagy and tumor development. Several reports indicate that inhibition of autophagy may be a promising approach for the treatment of BCR::ABL1-mediated leukemia [40, 41]. In this study, we demonstrate that PTBP2 binds to a subset of mRNAs with a very high affinity, of which BNIP3 is one, and was found to be stabilized by PTBP2. Despite its pro-death activity, BNIP3 expression in cancer often predicts an aggressive disease. Upregulation of BNIP3 characterizes cancer cell subpopulations with increased fitness and proliferation [42]. We also observed a strong correlation between Ptbp2 and Bnip3 expression in CML patients. The induction of autophagy through BNIP3 in pre-invasive breast cancers provides tumor cells with extra nutrients and promotes tumor progression [43]. Mechanistically, the LC3-II/LC3-I ratio decreased when PTBP2 was knocked out and increased when BNIP3 was overexpressed in the knockout cells. It was observed that the expression of Beclin-1 and ATG7 decreased in KO cells and also in KCL22-NTC (siRNA BNIP3) cells. We previously showed the antagonistic expression of PTBP1 and PTBP2 in CML cells [17]. Thus, consistent with our findings, the knockdown of PTBP1 (which increases the level of PTBP2) has been reported to lead to autophagy in colorectal and bladder cancer [44]. Also, the knockdown of PTBP1 caused the transition of LC3-I to LC3-II in breast and bladder cancer cells, while overexpression of PTBP1 reduced the transition of LC3-I to LC3-II [44, 45]. Fission is followed by selective fusion that segregates dysfunctional mitochondria and permits their removal by autophagy [46]. These reports and our data strongly suggest that the PTBP2-BNIP3 axis can positively regulate the proliferation of CML cells through the combinatorial action of mitochondrial fusion and the transition of LC3-I to LC3-II, thereby promoting autophagy. PTBP2-mediated induction of autophagy through BNIP3 may provide CML cells with extra nutrients and promote further tumor progression. The formation of large tumors with microscopic features of cellular pleomorphism and anaplasia and increased Ki-67 expression in mice injected with KCL22-NTC cells in comparison to Ptbp2-KO-KCL22 xenografts provide convincing evidence that the presence of PTBP2 simultaneously promotes proliferation, leading to aggressive tumor formation. CML is known to cause extramedullary hematopoiesis, particularly in organs like the spleen and liver. Enhanced engraftment and extramedullary infiltration in the transplantation model indicate that PTBP2 contributes to more aggressive disease, making it a potential marker for advanced-stage CML or blast crisis. Thus, targeting PTBP2-BNIP3-mediated autophagy by genetic ablation of PTBP2 or pharmacological inhibition of PTBP2 can reduce the disease severity in the CML cells.

## Supplementary information

Supplementary 1. Material and methods and 2. Figure legends

Supplementary Fig. 1

Supplementary Fig. 2

Supplementary Fig. 3

Supplementary Fig. 4

Supplementary Fig. 5

Figure WB Raw data

## Data Availability

The data supporting this study’s findings are available from the corresponding author upon request.
