# Supplementary figures and images for: PTBP2 promotes cell survival and autophagy in chronic myeloid leukemia by stabilizing BNIP3
Source: Cell Death Dis. 2025 Mar 20;16(1):195. doi: 10.1038/s41419-025-07529-9 (PMC11926076; doi:10.1038/s41419-025-07529-9)

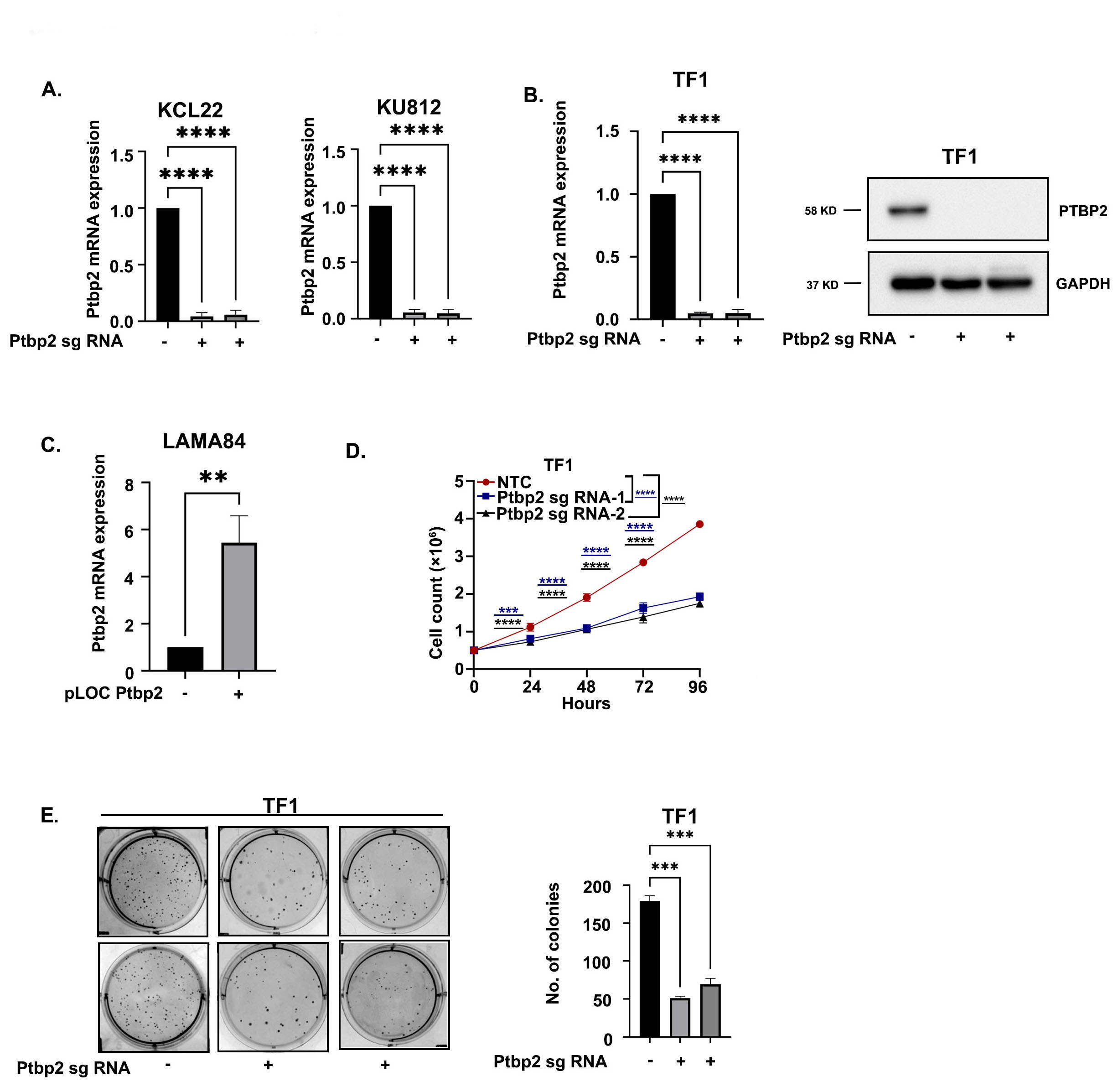

Supplement: Supplementary file 2 — Supplementary Fig. 1 [file 41419_2025_7529_MOESM2_ESM.jpg]

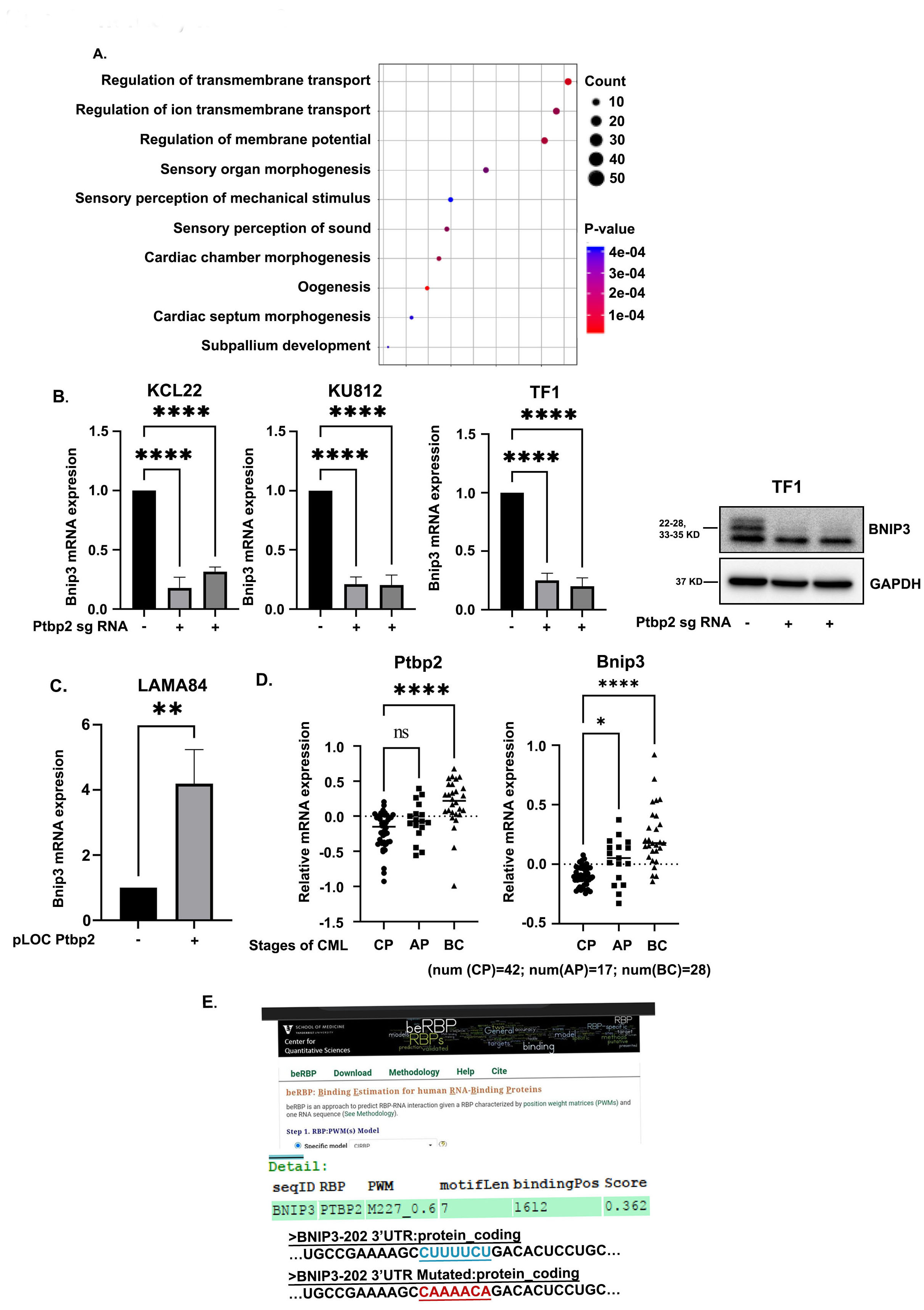

Supplement: Supplementary file 3 — Supplementary Fig. 2 [file 41419_2025_7529_MOESM3_ESM.jpg]

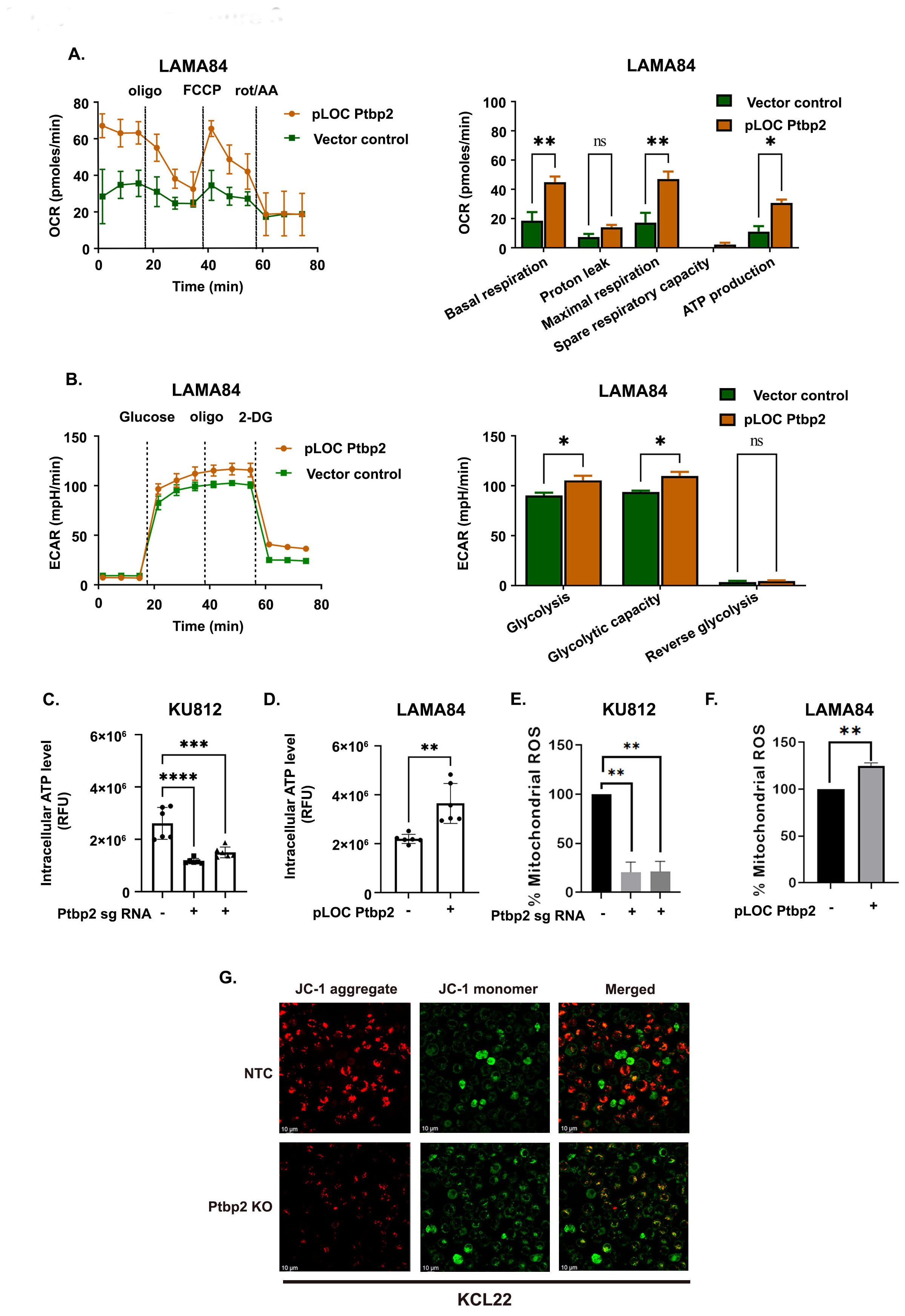

Supplement: Supplementary file 4 — Supplementary Fig. 3 [file 41419_2025_7529_MOESM4_ESM.jpg]

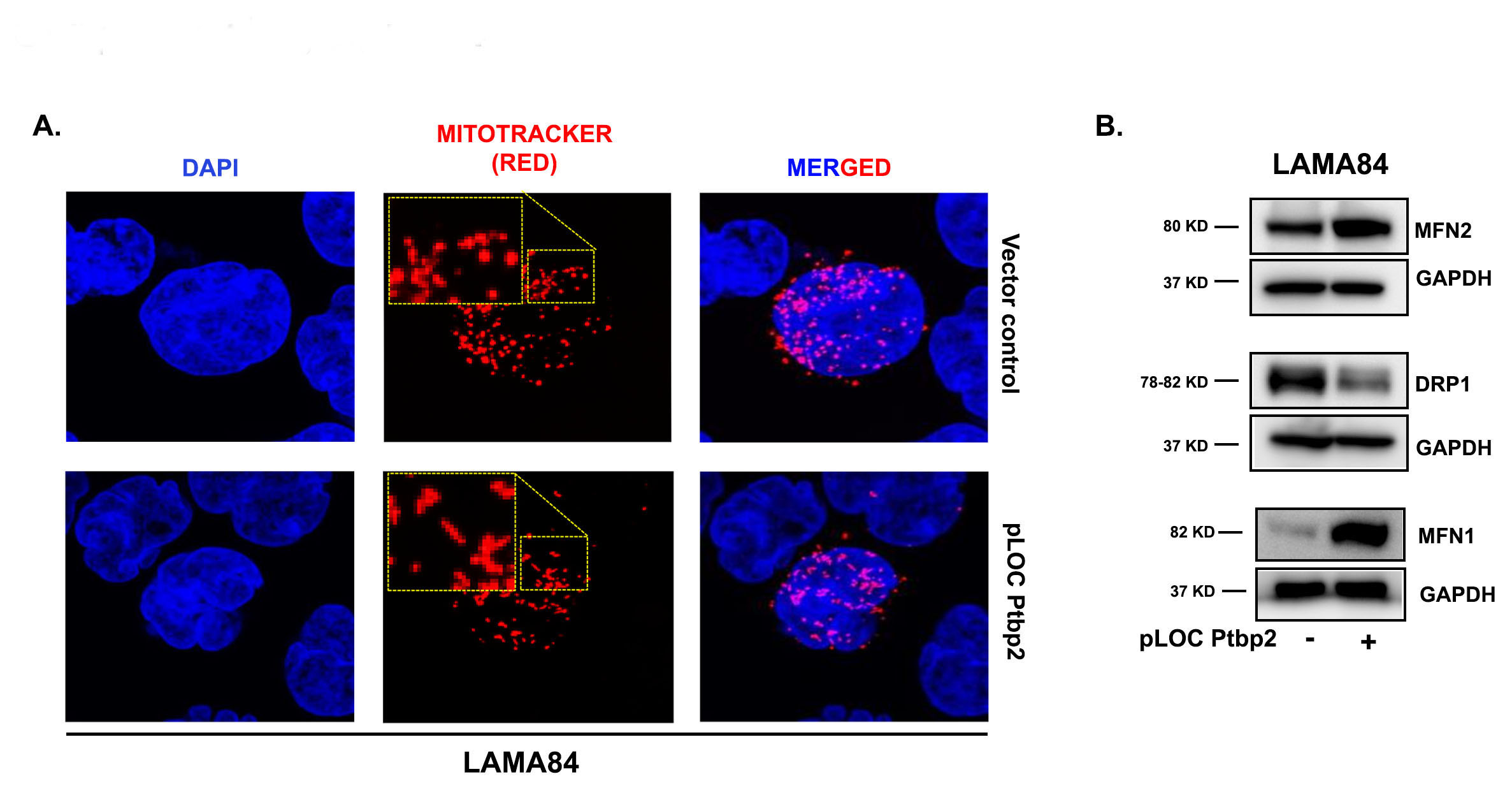

Supplement: Supplementary file 5 — Supplementary Fig. 4 [file 41419_2025_7529_MOESM5_ESM.jpg]

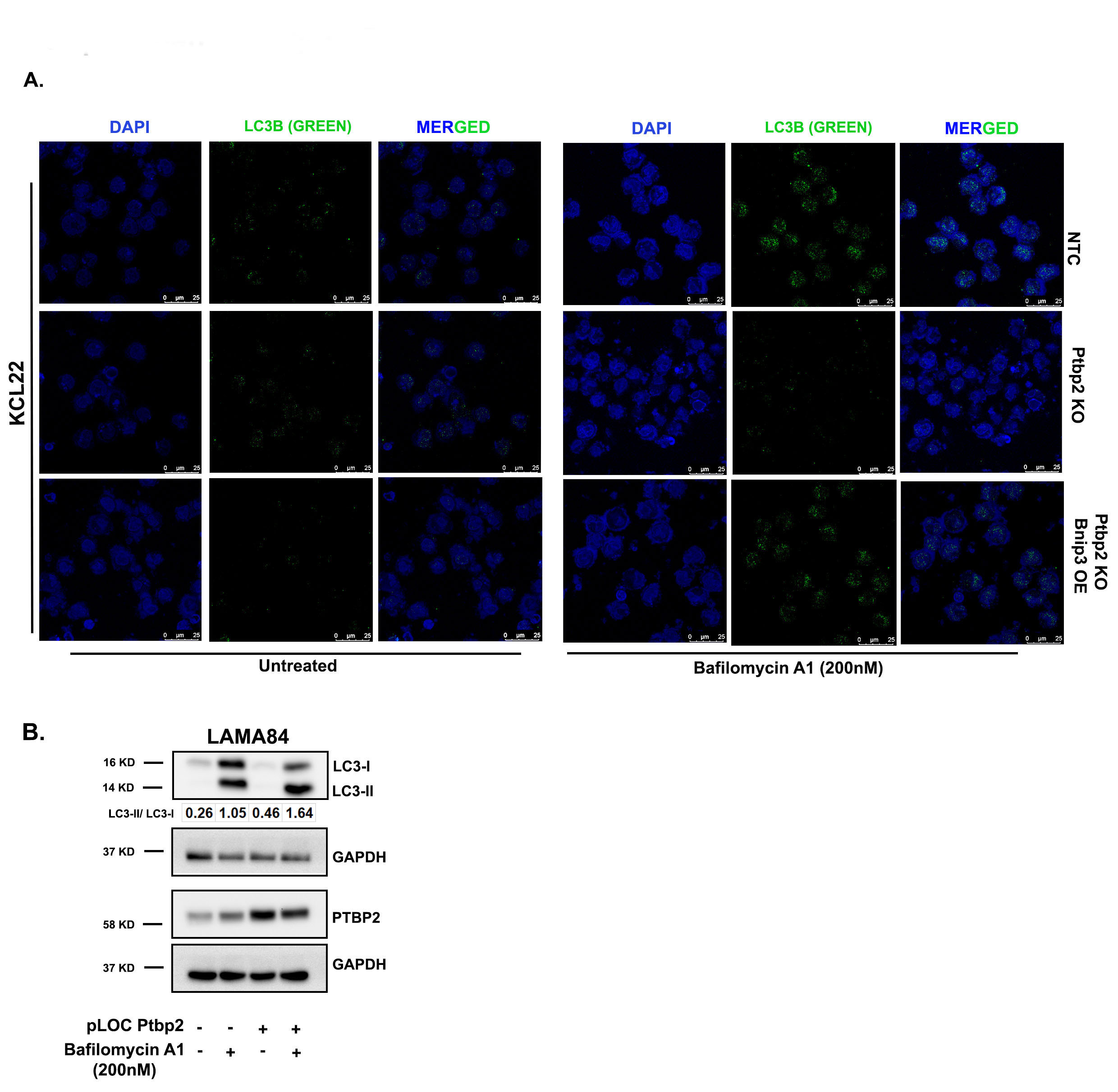

Supplement: Supplementary file 6 — Supplementary Fig. 5 [file 41419_2025_7529_MOESM6_ESM.jpg]
