# Supplementary material for: PTBP2 promotes cell survival and autophagy in chronic myeloid leukemia by stabilizing BNIP3
Source: Cell Death Dis. 2025 Mar 20;16(1):195. doi: 10.1038/s41419-025-07529-9 (PMC11926076; doi:10.1038/s41419-025-07529-9)

Figure 1

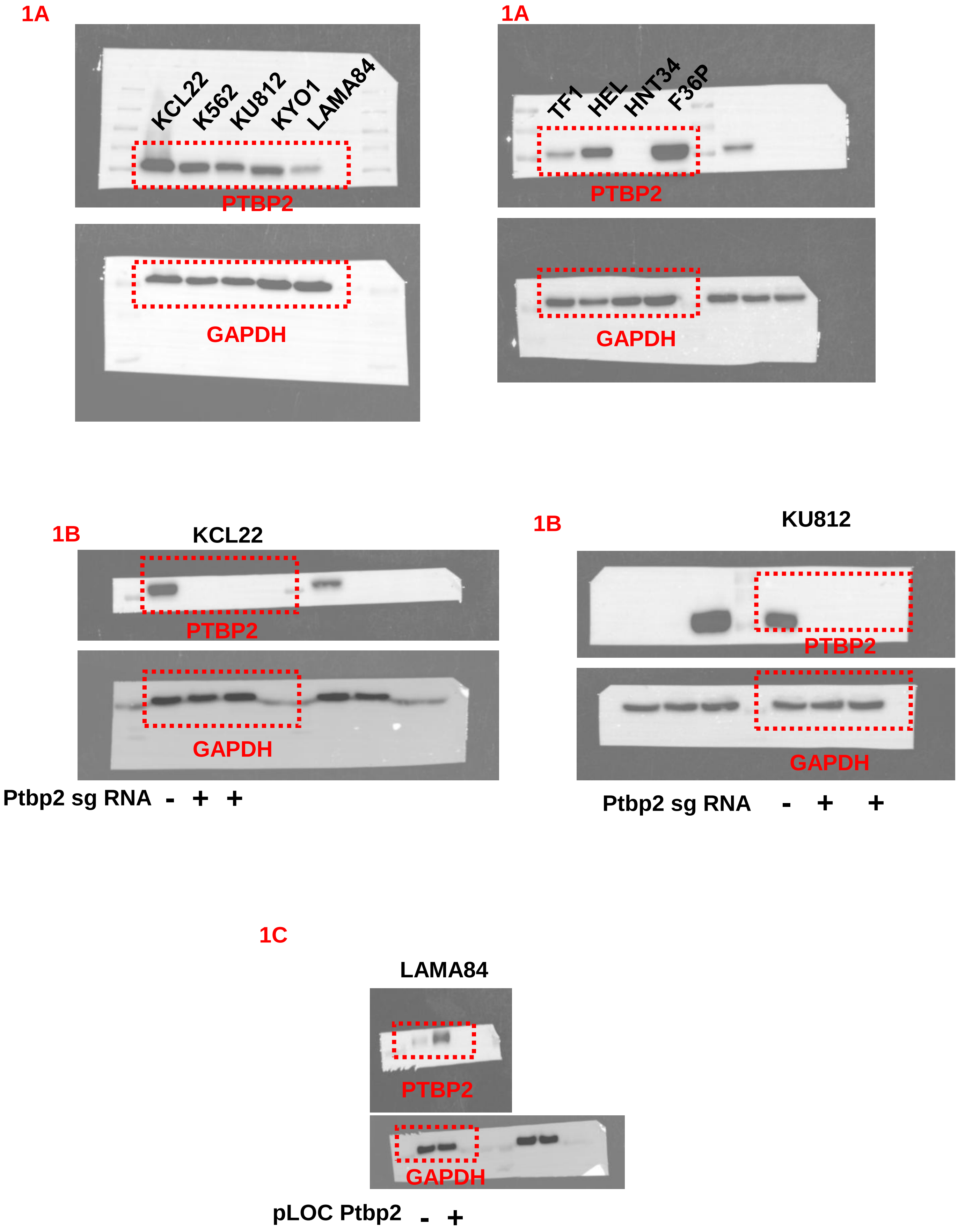
1A
KCL22
K562
KU812
KYO1
LAMA84
PTBP2
GAPDH
1A
TF1
HEL
HNT34
F36P
PTBP2
GAPDH
1B
KCL22
PTBP2
GAPDH
Ptbp2 sg RNA - + +
1B
KU812
PTBP2
GAPDH
Ptbp2 sg RNA - + +
1C
LAMA84
PTBP2
GAPDH
pLOC Ptbp2 - +

Figure 2

2A **KCL22**

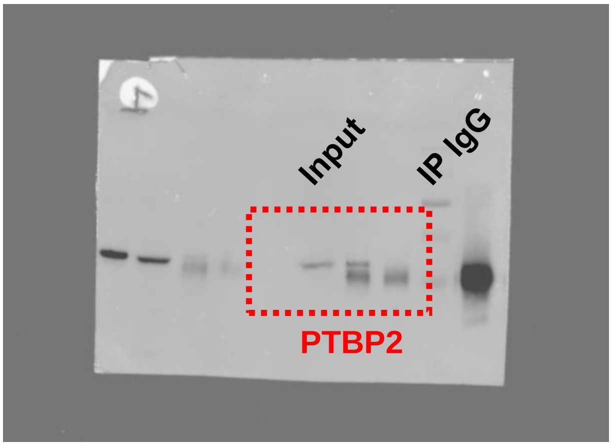

2F **KU812**

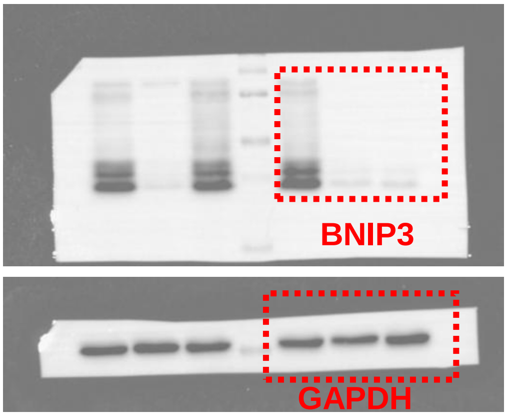

Ptbp2 sg RNA **- + +**

2F **KCL22**

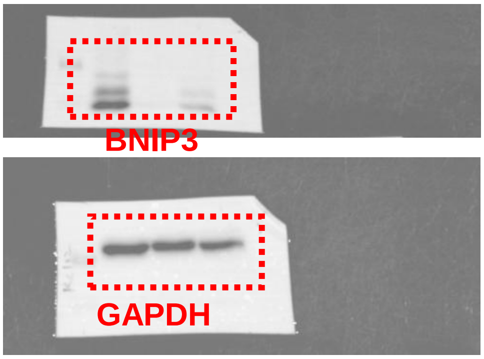

Ptbp2 sg RNA **- + +**

2G

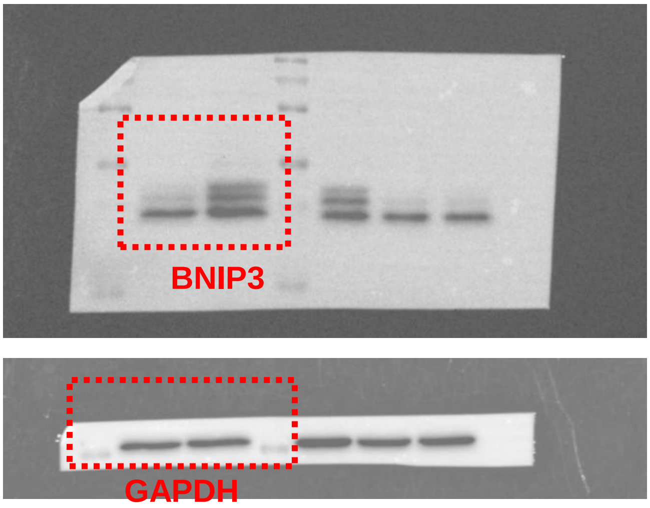

pLOC Ptbp2 **- +**

2I

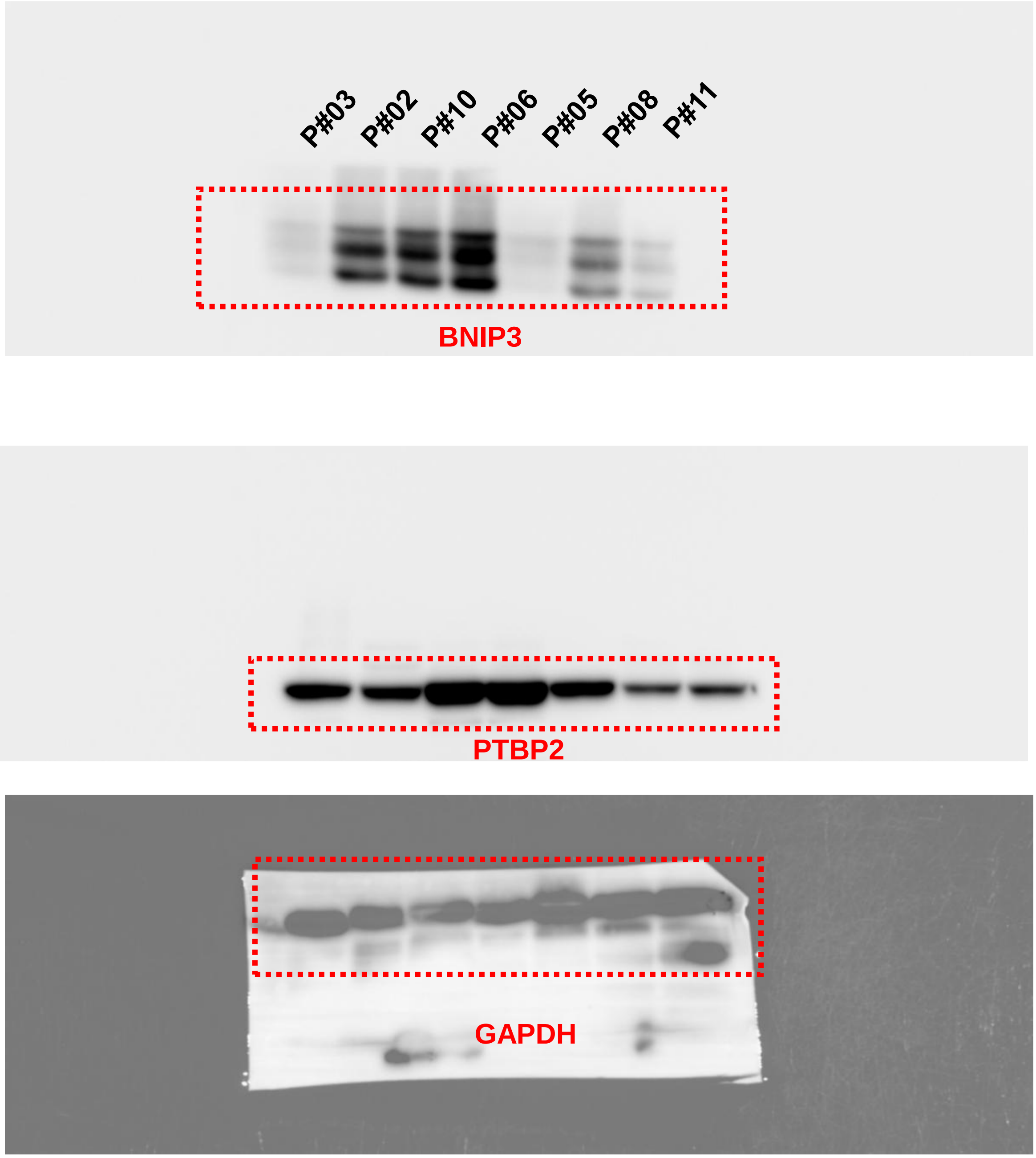

Figure 4

4B

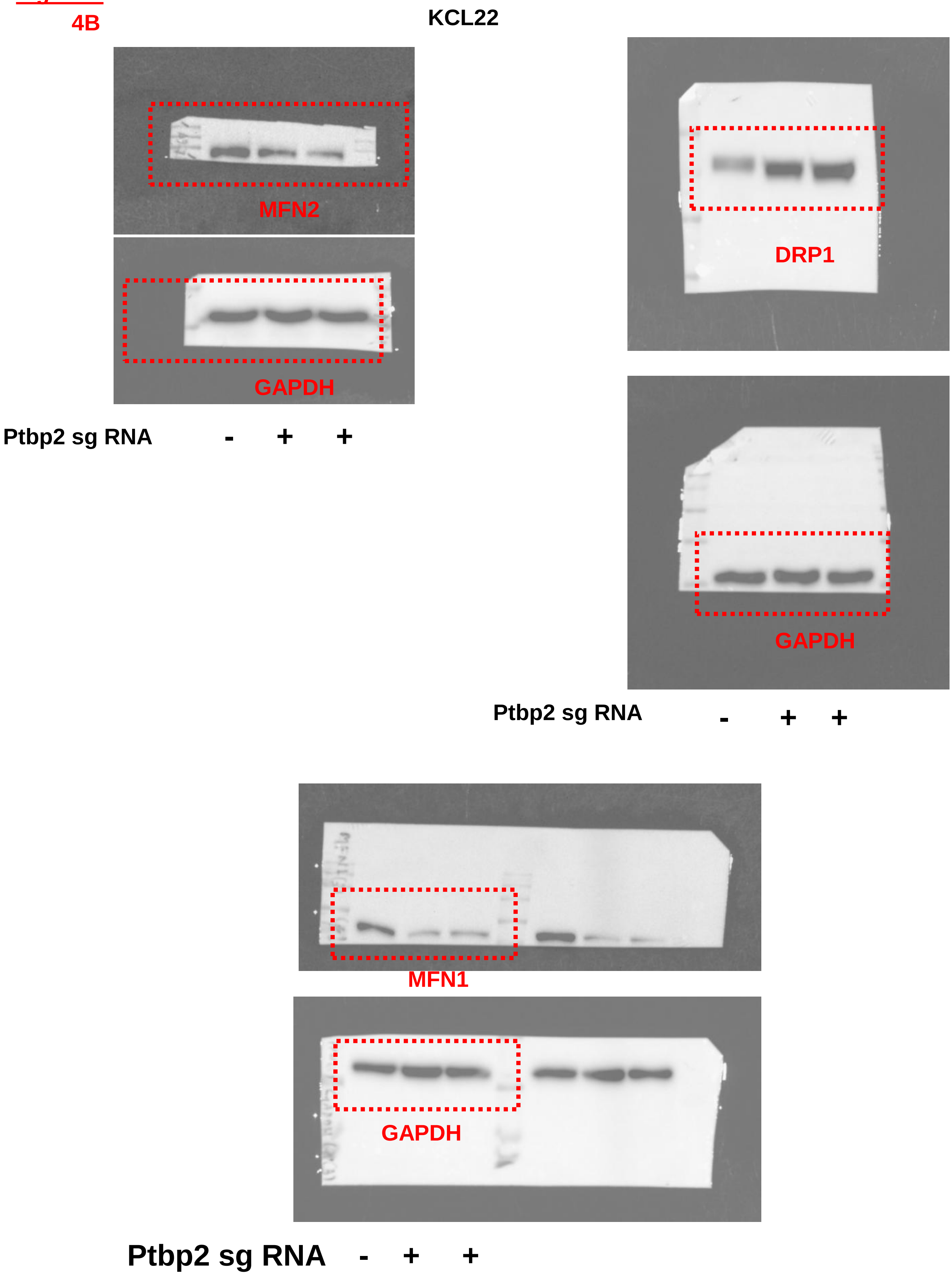
KCL22
MFN2
GAPDH
Ptbp2 sg RNA - + +
DRP1
GAPDH
Ptbp2 sg RNA - + +
MFN1
GAPDH
Ptbp2 sg RNA - + +

Figure 5

5A

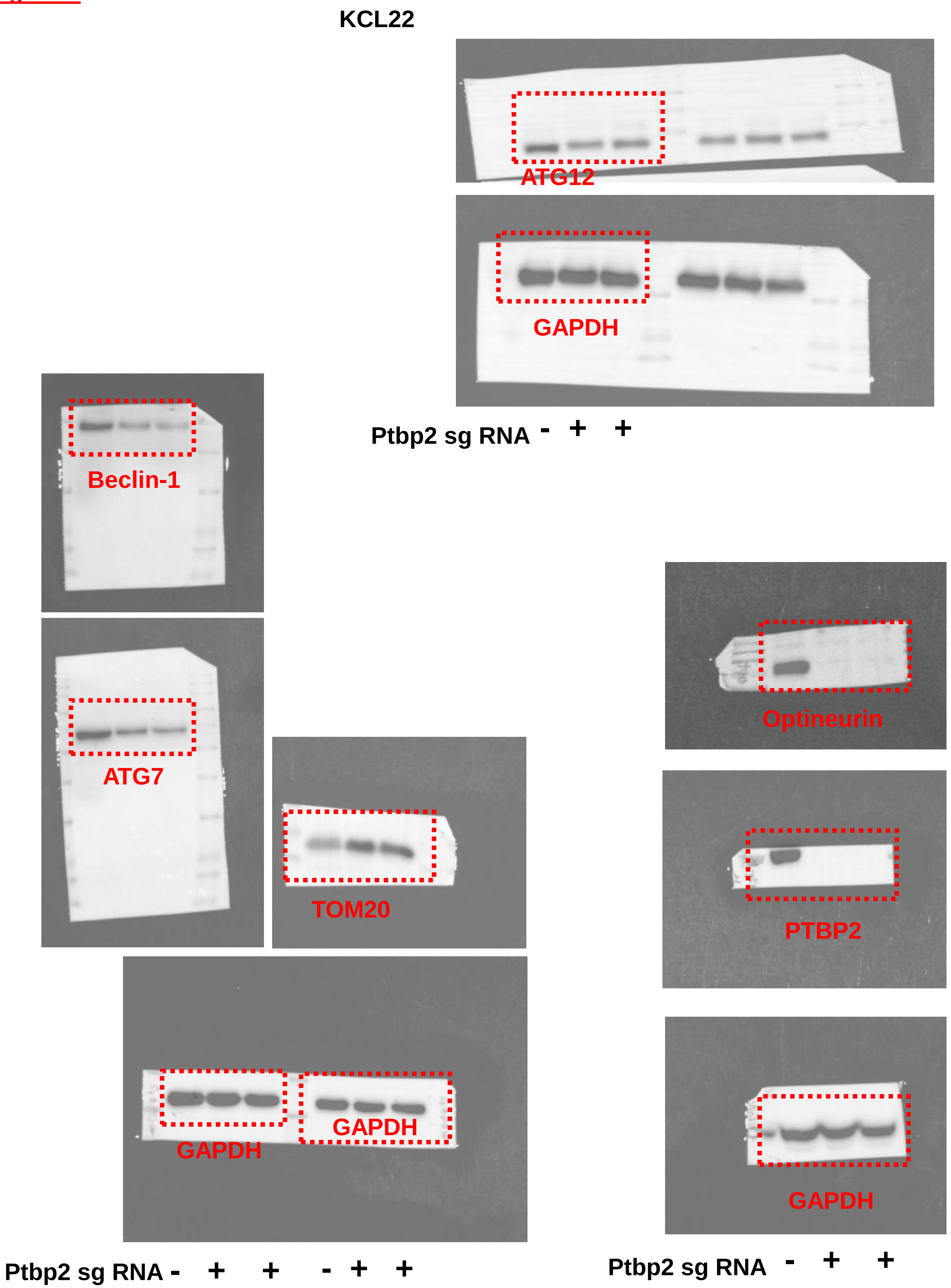

KCL22
ATG12
GAPDH
Ptbp2 sg RNA - + +
Beclin-1
ATG7
TOM20
Optineurin
PTBP2
GAPDH
GAPDH
GAPDH
Ptbp2 sg RNA - + + - + +
Ptbp2 sg RNA - + +

KU812

5A

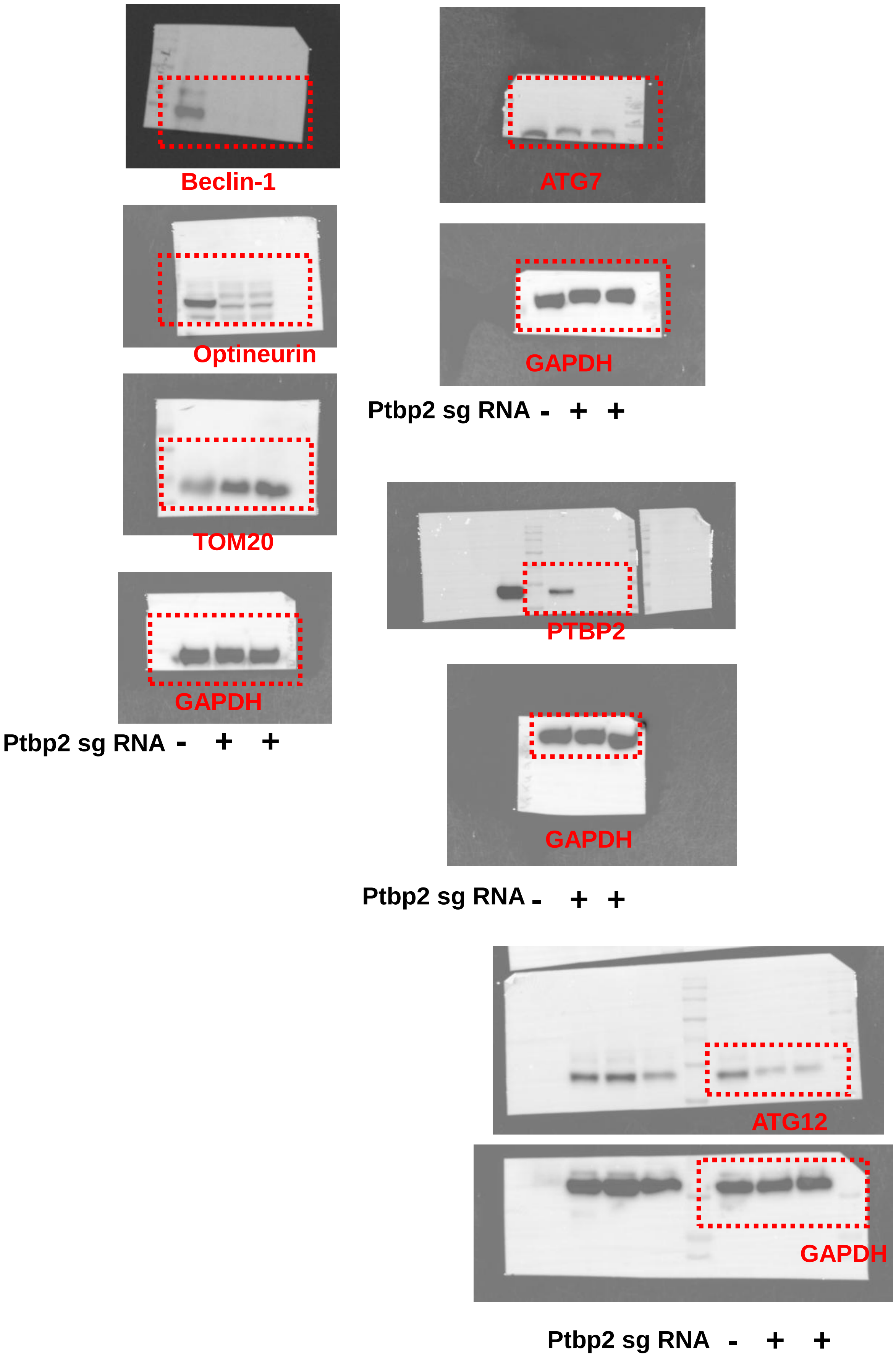
Beclin-1
ATG7
Optineurin
GAPDH
Ptbp2 sg RNA - + +
TOM20
PTBP2
GAPDH
Ptbp2 sg RNA - + +
GAPDH
Ptbp2 sg RNA - + +
ATG12
GAPDH
Ptbp2 sg RNA - + +

5B

LAMA84

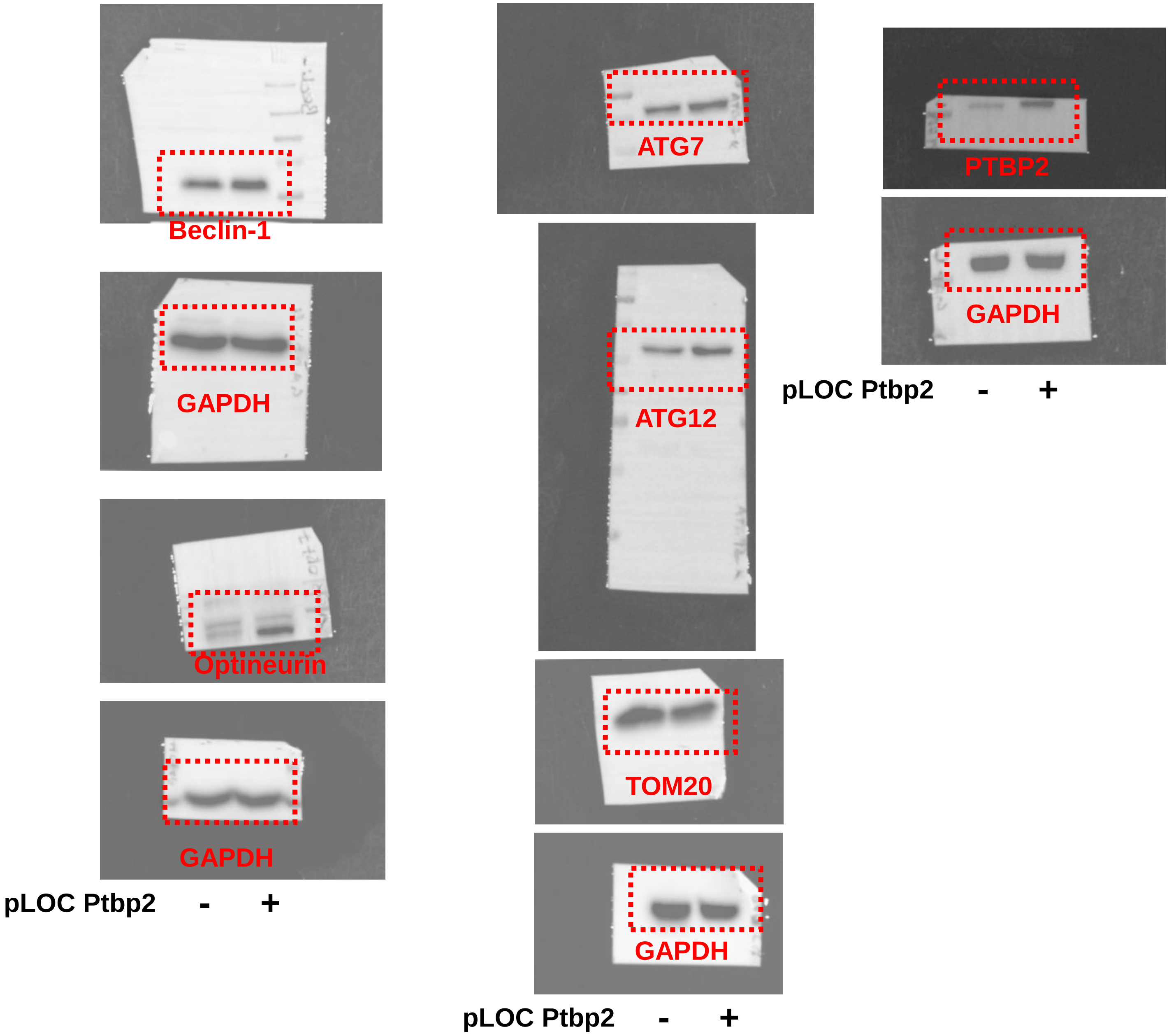
Beclin-1
GAPDH
Optineurin
GAPDH
pLOC Ptbp2 - +
ATG7
ATG12
TOM20
GAPDH
pLOC Ptbp2 - +
PTBP2
GAPDH
pLOC Ptbp2 - +

5C

KCL22

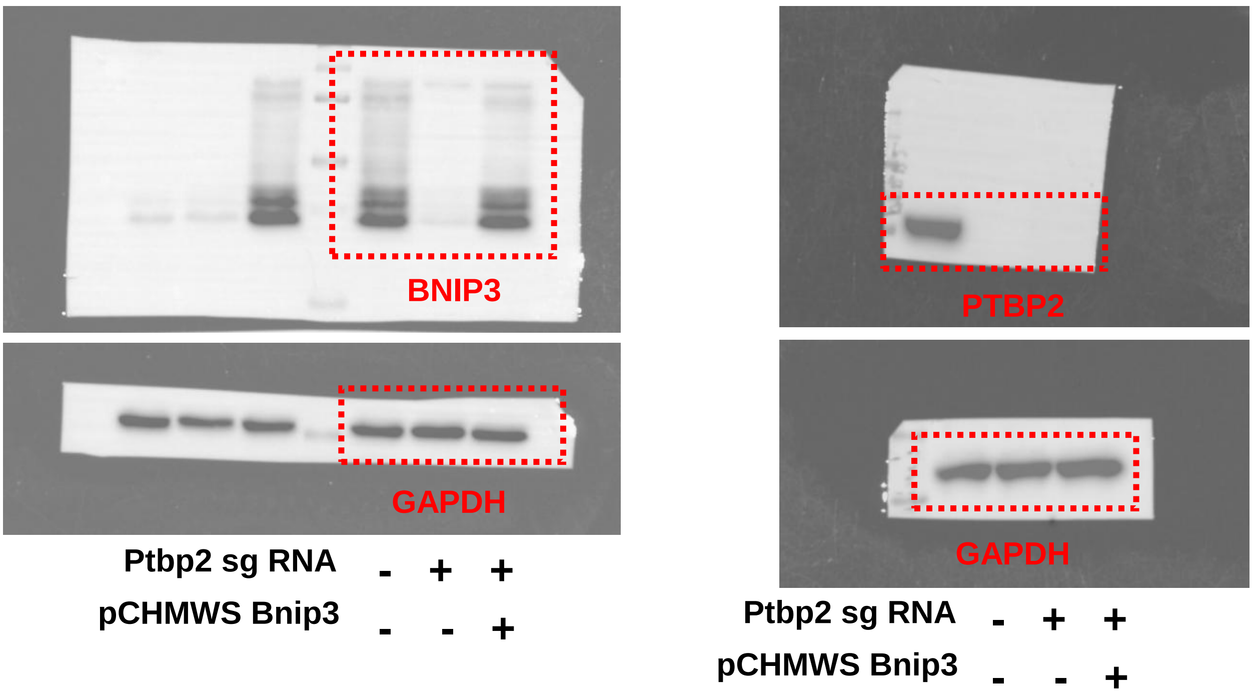

BNIP3
GAPDH
Ptbp2 sg RNA - + +
pCHMWS Bnip3 - - +
PTBP2
GAPDH
Ptbp2 sg RNA - + +
pCHMWS Bnip3 - - +

5D

KCL22

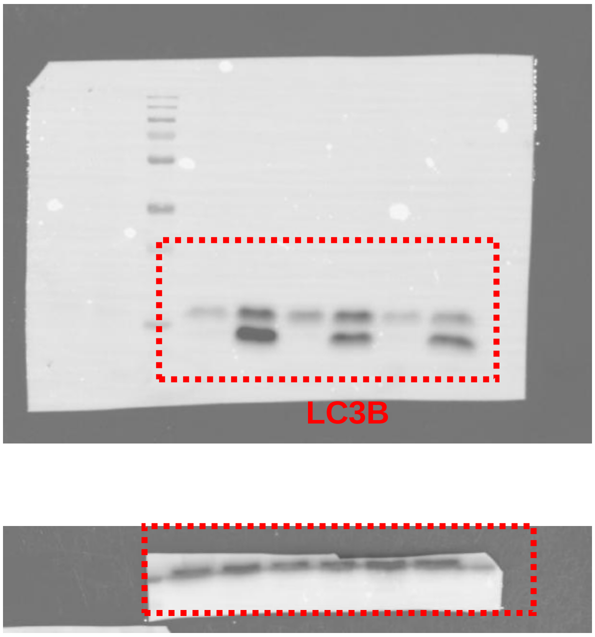

GAPDH

Ptbp2 sg RNA	-	-	+	+	+	+
pCHMWS Bnip3	-	-	-	-	+	+
Bafilomycin A1 (200nM)	-	+	-	+	-	+

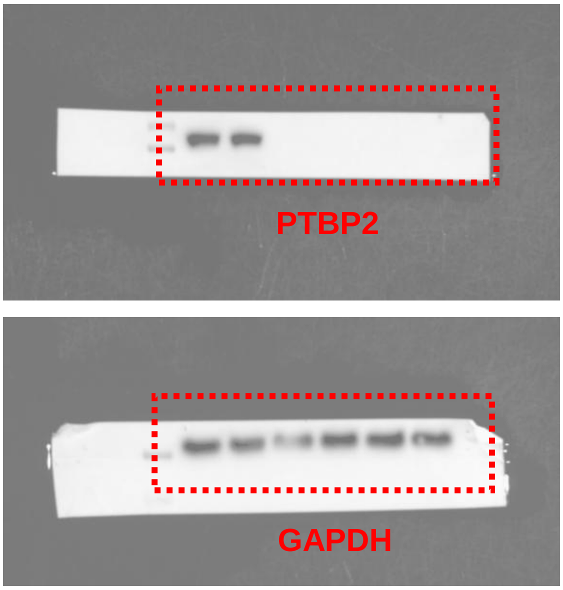

Ptbp2 sg RNA	-	-	+	+	+	+
pCHMWS Bnip3	-	-	-	-	+	+
Bafilomycin A1 (200nM)	-	+	-	+	-	+

KCL22

5G

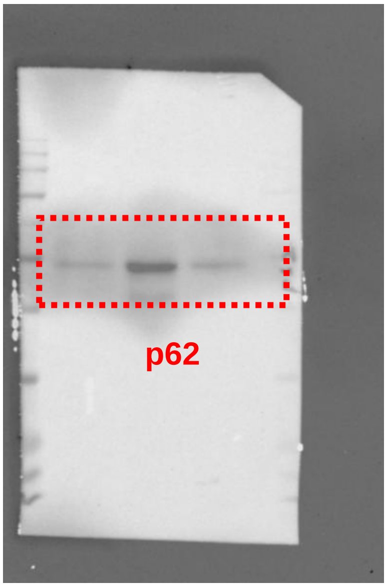

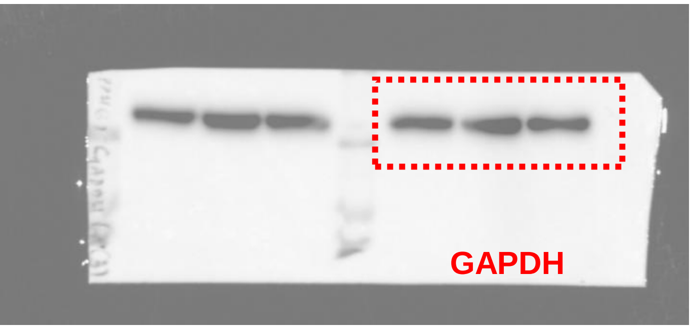

Ptbp2 sg RNA	-	+	+
pCHMWS Bnip3	-	-	+

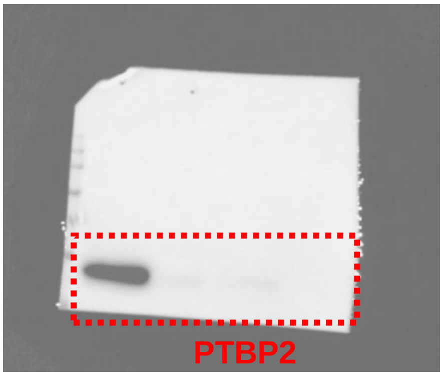

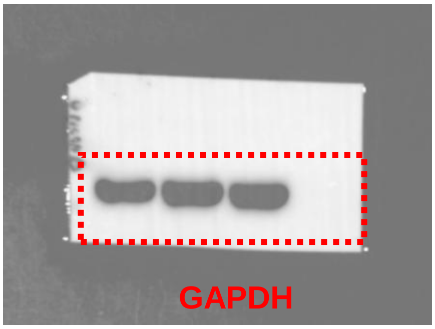

Ptbp2 sg RNA	-	+	+
pCHMWS Bnip3	-	-	+

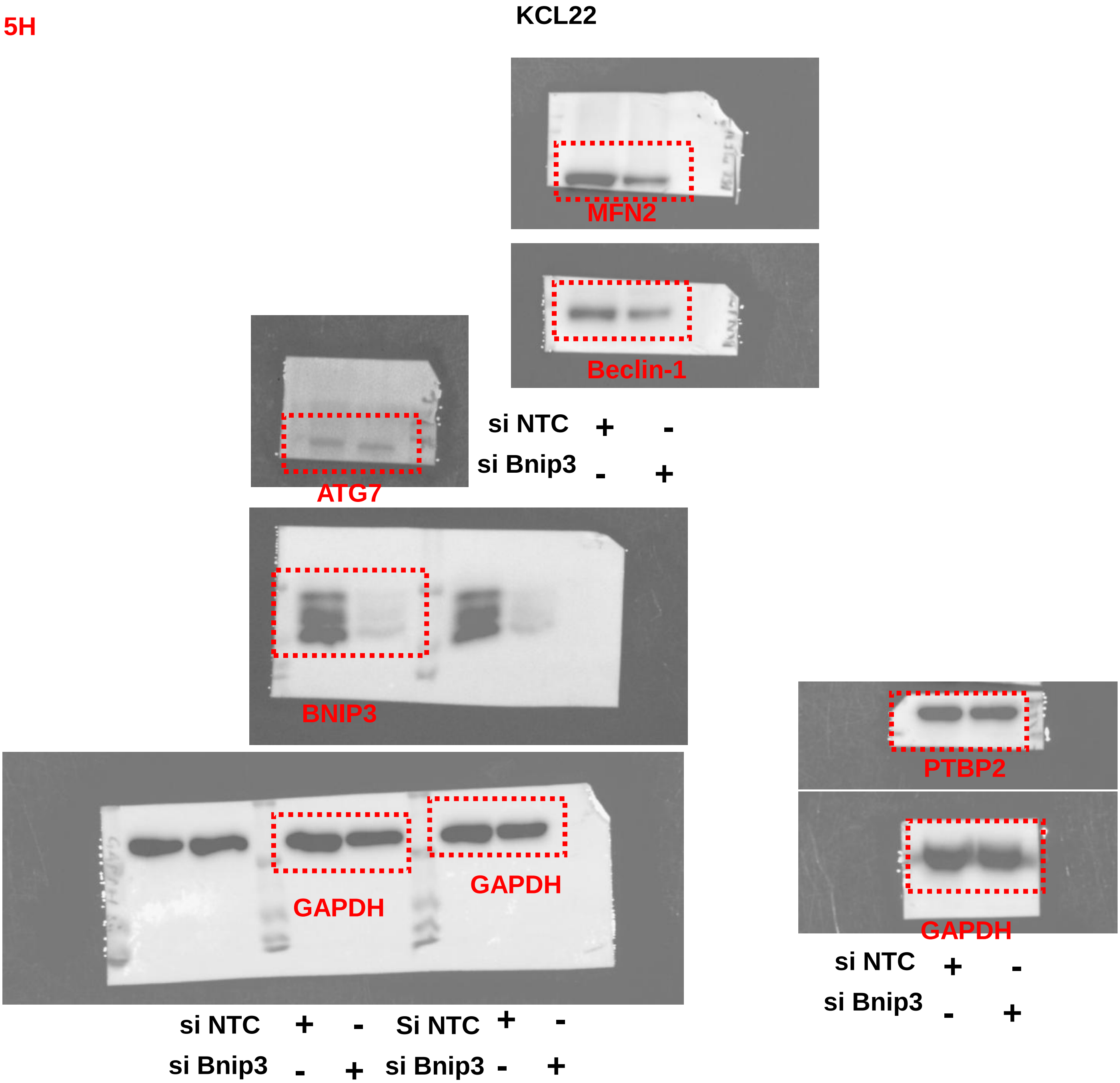
5H
KCL22
MFN2
Beclin-1
ATG7
si NTC + -
si Bnip3 - +
BNIP3
GAPDH
GAPDH
si NTC + -
si Bnip3 - +
Si NTC + -
si Bnip3 - +
PTBP2
GAPDH
si NTC + -
si Bnip3 - +

5J

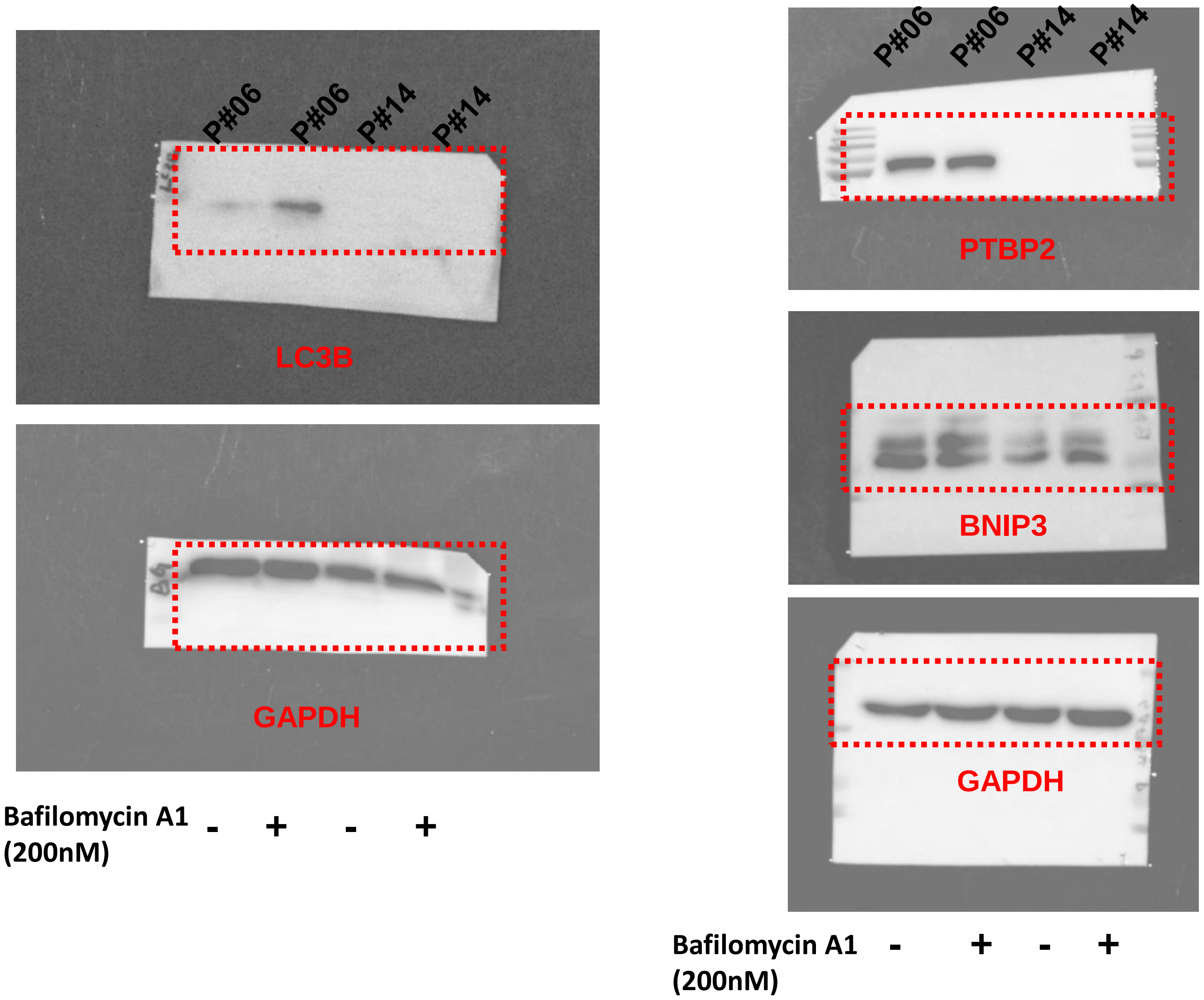

P#06
P#06
P#14
P#14
LC3B
GAPDH
Bafilomycin A1 (200nM) - + - +
P#06
P#06
P#14
P#14
PTBP2
BNIP3
GAPDH
Bafilomycin A1 (200nM) - + - +

Supplementary figure 1

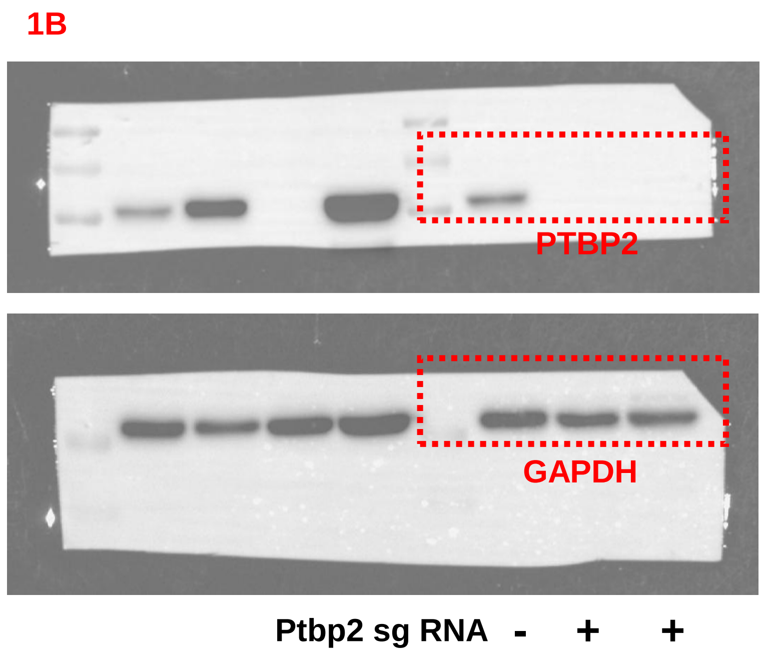

Supplementary figure 2

2B

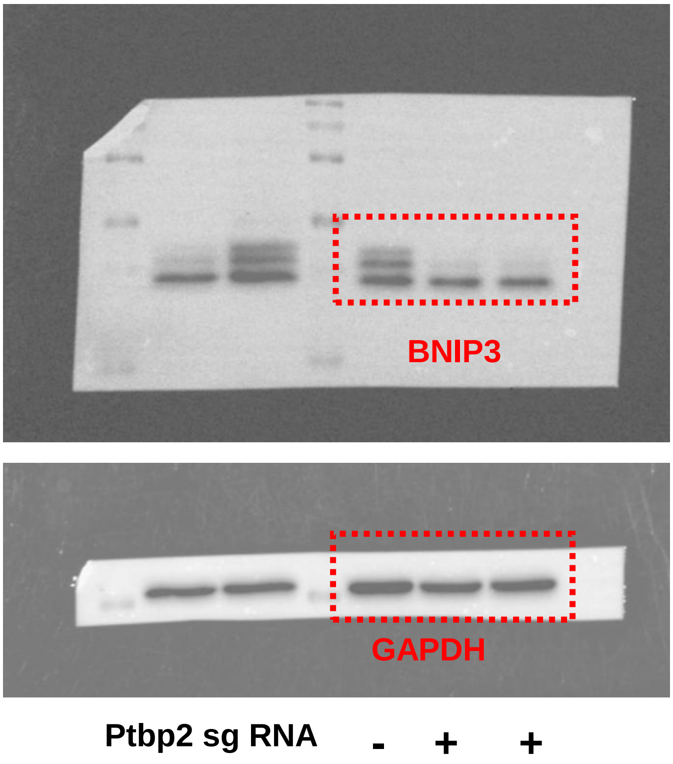

Supplementary figure 4

4B

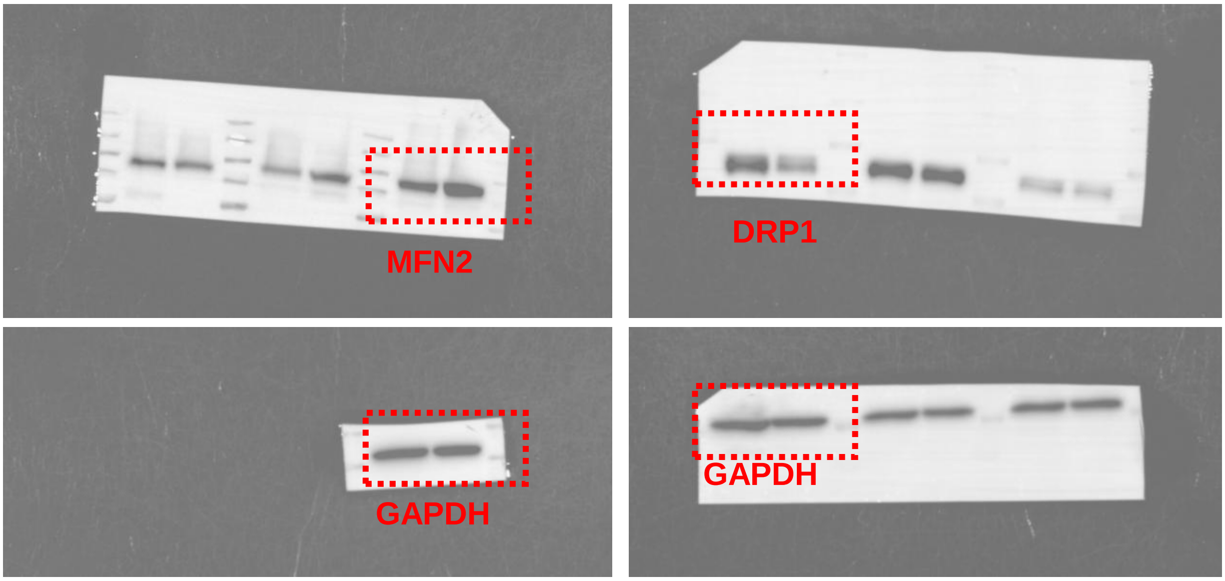

pLOC Ptbp2 - + - +

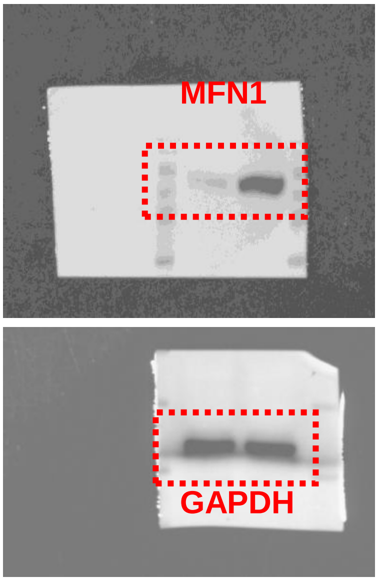

pLOC Ptbp2 - +

Supplementary figure 5

5B

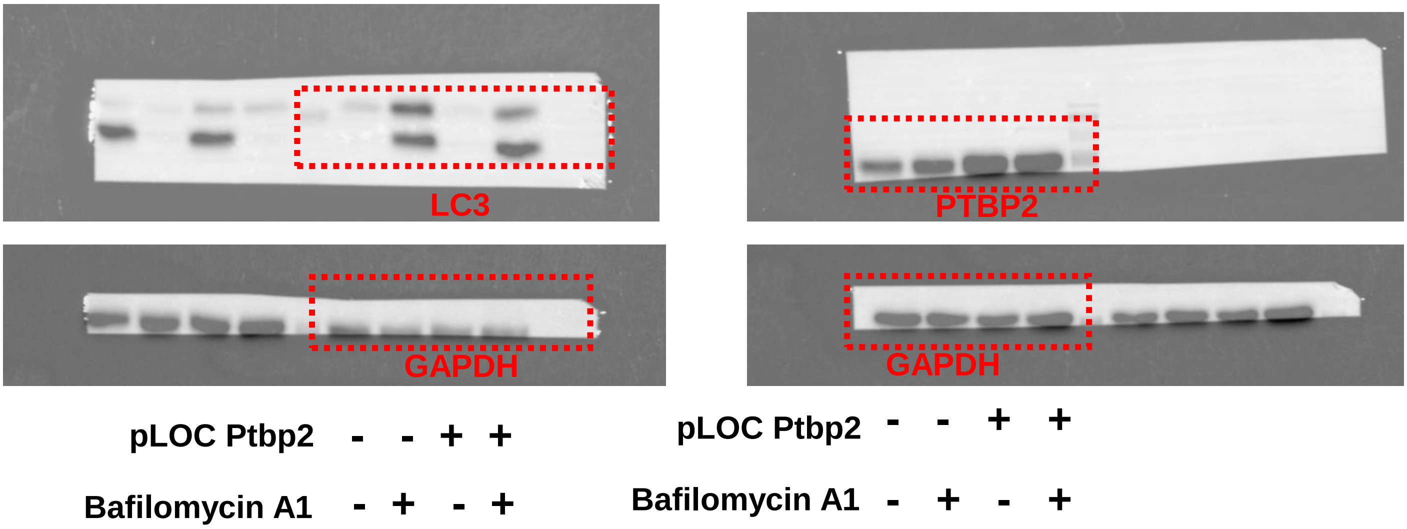

Supplement: Supplementary file 7 — Figure WB Raw data [file 41419_2025_7529_MOESM7_ESM.pdf]
